# Effectiveness of Neural Mobilisation on Pain Intensity, Functional Status, and Physical Performance in Adults with Musculoskeletal Pain – A Systematic Review with Meta-Analysis

**DOI:** 10.1177/02692155231215216

**Published:** 2023-11-21

**Authors:** Frederico Mesquita Baptista, Ellen Nery, Eduardo Brazete Cruz, Vera Afreixo, Anabela G Silva

**Affiliations:** 1 CINTESIS.UA@RISE, Department of Medical Sciences, 56062 University of Aveiro, Aveiro, Portugal; 2 Departamento Fisioterapia, 70869 Instituto Politécnico de Setúbal, Escola Superior de Saúde, Setúbal, Portugal / CHRC – Comprehensive Health Research Center, Setubal, Portugal; 3 Department of Mathematics, CIDMA – Center for Research and Development in Mathematics and Applications, 56062 University of Aveiro, Aveiro, Portugal; 4 CINTESIS.UA@RISE, School of Health Sciences, 56062 University of Aveiro, Aveiro, Portugal

**Keywords:** Physical therapy, neural mobilisation, musculoskeletal disorders, pain, disability

## Abstract

**Objective:**

To investigate up-to-date evidence of the effectiveness of neural mobilisation techniques compared with any type of comparator in improving pain, function, and physical performance in people with musculoskeletal pain.

**Data sources:**

The following sources were consulted: PubMed, Web of Science, CENTRAL, CINAHL, Scopus, and PEDro databases; scientific repositories; and clinical trial registers. The last search was performed on 01/06/2023.

**Methods:**

Two reviewers independently assessed the studies for inclusion. We included randomised, quasi-randomised, and crossover trials on musculoskeletal pain in which at least one group received neural mobilisation (alone or as part of multimodal interventions). Meta-analyses were performed where possible. The RoB 2 and the Grading of Recommendations Assessment, Development and Evaluation tools were used to assess risk of bias and to rate the certainty of evidence, respectively.

**Results:**

Thirty-nine trials were identified. There was a significant effect favouring neural mobilisation for pain and function in people with low back pain, but not for flexibility. For neck pain, there was a significant effect favouring neural mobilisation as part of multimodal interventions for pain, but not for function and range of motion. Regarding other musculoskeletal conditions, it was not possible to conclude whether neural mobilisation is effective in improving pain and function. There was very low confidence for all effect estimates.

**Conclusions:**

Neural mobilisation as part of multimodal interventions appears to have a positive effect on pain for patients with low back pain and neck pain and on function in people with low back pain. For the other musculoskeletal conditions, results are inconclusive.

## Introduction

Musculoskeletal conditions are related to impairments in muscles, bones, joints, and adjacent connective tissues, and are a major contributor to disability worldwide, affecting approximately 1.71 billion people.^1^ The most common symptom of musculoskeletal conditions is pain, which can be acute/subacute (short term) or chronic (long term).^1^ Some aspects of physical performance can also be altered, such as muscle strength, gait velocity, and motor control, considering the motor adaptations due to the experience of pain.^2^ The Global Burden of Disease 2019 showed that musculoskeletal conditions are responsible for approximately 17% of the total years lived with disability, with low back and neck pain together representing more than half of this percentage (9.97%).^3^ The most recent data indicate a prevalence of 570 (7350 per 100,000) and 220 million (2880 per 100,000) of people suffering from low back and neck pain worldwide, respectively.^4^ In addition, musculoskeletal conditions are among the most common and costly work-related health problems in the United States of America and European Union,^5,6^ corresponding to a cost of U$ 980 billion in 2014 (5.76% of the annual Gross Domestic Product) in the United States of America^6^ and EUR 30.4 billion in 2016 in Germany (1.0% of the annual Gross Domestic Product), for example.^5^

There are many intervention strategies that can be used to treat signs and symptoms of musculoskeletal conditions, such as patient education, non-pharmacological techniques, pharmacological approaches, and, ultimately, surgery.^7^ However, non-pharmacological strategies are preferred to minimise the use of pharmacotherapy,^7^ where *neural mobilisation techniques* appear as one of the interventions widely used by physiotherapists.^8,9^

Neural mobilisation techniques comprise a combination of joint movements that promote sliding or tensioning of the structures that involve the nerve, the nerve itself and/or the central nervous tissues.^10–12^ When the joints are moved in such a way that neural tissue elongation at a given joint is simultaneously relieved by a reduction in tissue length at another joint, the technique is defined as a *sliding technique.*^10^ On the other hand, a *tensioning technique* occurs when there is a displacement of both neural tissue endings in opposite directions or the displacement of one of the endings in a tensioned direction while the other remains fixed.^10^ *Sliding techniques* allow for more non-aggressive nerve excursion, allowing movement to be presented to the brain in different ways, disengaging learned expectations of pain.^10^ Larger amplitude movements tend to decrease fear of movement and may help with remapping altered representations.^10^ On the other hand, *tensioning techniques* seem to improve pressure pain thresholds^13^ and intraneural oedema dispersion^14^ when compared to *sliding techniques*.

Previous reviews have investigated the effectiveness of neural mobilisation mainly in nerve-related musculoskeletal disorders,^8,15–19^ but evidence on its effectiveness in musculoskeletal pain conditions without neurological impairments, as well as in aspects of physical performance, is sparse. Thus, this systematic review aims to investigate up-to-date evidence on the effectiveness of neural mobilisation techniques in improving pain, function, and physical performance in people with different musculoskeletal pain conditions without neurological deficits.

## Methods

This systematic review was performed following the guidelines of the Cochrane Handbook^20^ and Preferred Reporting Items for Systematic Reviews and Meta-Analysis (PRISMA).^21,22^ The protocol was registered in PROSPERO (CRD42021288387) and published elsewhere.^23^ All changes to the original protocol are justified in Supplemental File 1. The eligibility criteria were defined as follows:

- ***Population* –** People over 18 years old with musculoskeletal pain without neurological deficits**.** Trials involving participants with neurological impairment (motor and/or sensory) and with neuropathic descriptors were excluded (e.g., subjects with radicular symptoms [decreased reflexes, significant loss of strength or major changes in sensation, symptoms related to specific dermatomes and/or myotomes]; tunnel syndromes; diabetic neuropathy; spinal cord injury; stroke; neurodegenerative diseases; severe mechanosensitivity or alterations in nerve conduction [hypoesthesia or anaesthesia]). Trials involving post-surgical pain (e.g., spinal arthrodesis, breast cancer surgery) were also excluded, as well as participants with clinical signs or a diagnosis of infection diseases, tumour/cancer, severe depression, or other psychiatric disorders.
- ***Intervention* –** Neural mobilisation techniques used as a single intervention or as part of multimodal interventions.
- ***Comparisons* –** Both active (e.g., a different variant of neural mobilisation technique, other therapy) and inactive control interventions (e.g., sham therapy, no treatment).
- ***Outcomes* –** *Primary outcomes:* pain intensity and functional status. *Secondary outcomes:* health and skill-related components of physical fitness (flexibility, balance, and muscular strength), pressure pain threshold, conditioned pain modulation, data related to immune responses, and morphological and neurophysiological changes in peripheral nerves.
- ***Study design* –** Randomised, quasi-randomised, and crossover trials. The remaining study designs were excluded.

We searched for articles published until June 2023 in the data sources described in Table 1. The search was performed by one reviewer (FB). Full search strategies are detailed in Supplemental File 2.

**Table 1.** Research sources.

Bibliographic databases	PubMed Web of Science (five collections included – Web of Science Core Collection, Korean Journal Database, MEDLINE, Russian Science Citation Index and SciELO Citation Index) MEDLINE (via PubMed and Web of Science) Cochrane Central Register of Controlled Trials (CENTRAL) – accessed through EBSCO host Web Cumulative Index to Nursing and Allied Health Literature (CINAHL Plus with Full Text) – accessed through EBSCO host Web Scopus Physiotherapy Evidence Database (PEDro)
Scientific repositories	Open Access Scientific Repositories of Portugal (RCAAP – acronym in Portuguese)
Registers	ClinicalTrials.gov The International Clinical Trials Registry Platform of the World Health Organization
Other sources	Reference lists of the included studies and previous systematic reviews

All articles found were uploaded to an online evidence synthesis tool (CADIMA v. 2.2.3, Julius Kuehn-Institute [JKI], Federal Research Centre for Cultivated Plants, Quedlinburg, Germany, 2021) where duplicates were removed.^24,25^ Two reviewers (FB and EN) independently screened the titles and abstracts. The full reports were read by two of three reviewers (FB, AS and EC), also independently. All disagreements were resolved by a consensus.

One author (FB) performed the data collection. Bibliographic information, subgroups of patients, sociodemographic characteristics, outcome measures and their measurement instruments, evaluation period, and main results were collected. Authors were contacted by email when relevant data were not available in the article. Of 21 authors contacted, only 7 responded.

Participant and study design characteristics, sample sizes, summary statistics, effect estimates, and precision measures (when available) were extracted from the trials. The details of the interventions were obtained following the TIDieR guidelines.^26^

The RoB 2 and ROBINS-I tools were used to assess the risk of bias.^27–30^ Two of three authors (FB, AS and EC) independently performed the assessments and disagreements were resolved in consensus meetings. To obtain the general risk of bias judgment, the algorithm proposed by the tools was used. Confidence in the effect estimates was evaluated using the GRADE approach via the GRADEpro computer software (McMaster University, Ontario).^31^

Considering that multiple instruments were used to measure the same outcome domain between trials, we used the standardised mean difference (Cohen´s d) effect sizes with their respective 95% confidence intervals as the effect measure for continuous outcomes. For studies that did not enter meta-analyses, we calculated mean differences between groups, p-values, effect sizes, and confidence intervals, where possible (Supplemental File 3). Cohen´s d was classified as: small (d = 0.20), medium (d = 0.50), and large (d = 0.80).^32^

Studies were grouped considering specific musculoskeletal conditions and the outcomes assessed at post-intervention. In situations where two or more studies reported sufficient data, results were combined through meta-analyses,^33^ otherwise a descriptive approach was used.

Pre- and post-intervention mean values and standard deviations were used to calculate effect sizes and conversions from other statistical parameters were performed as detailed in the literature.^34–36^ When necessary, data of two or more groups were combined using the formula proposed by the Cochrane guidelines.^37^

All analyses were performed using the R Version 2022.07.2 Build 576 (R Core Team, Vienna),^38^ through the functions esc_mean_sd {esc} – to calculate effect sizes; metagen {meta} – to perform meta-analyses. The random effects model was chosen considering the high methodological heterogeneity between trials. To summarise the overall effect size, the weighted average was used through the inverse variance method. To estimate Tau, the restricted maximum-likelihood method was used.^39^ The heterogeneity was assessed using Cochran´s Q test (Chi-Square Test) and quantified by I^2^ Higgins´ and Tau statistics.^40^ The significance value considered for the Chi-Square Test was 0.1 as suggested in the literature.^41^ The degree of heterogeneity using I^2^ Higgins´ statistics was classified as following: (1) low (0% to 25%), (2) moderate (25% to 50%), and (3) high (above 50%).

Subgroup analyses considering the type of intervention used (*single intervention* vs. *multimodal intervention* and *sliding technique* vs. *tensioning technique*) were performed to explore possible causes of heterogeneity between trials.

When 10 or more studies were included in the meta-analysis, the assessment of publication bias was performed using funnel plots.^42^ Egger and Begg tests were applied to test the symmetry of the funnel plot.^43–45^ P-curve analysis was carried out to assess whether publication bias was generated due to selective reporting.^46–48^

## Results

**Figure 1.**
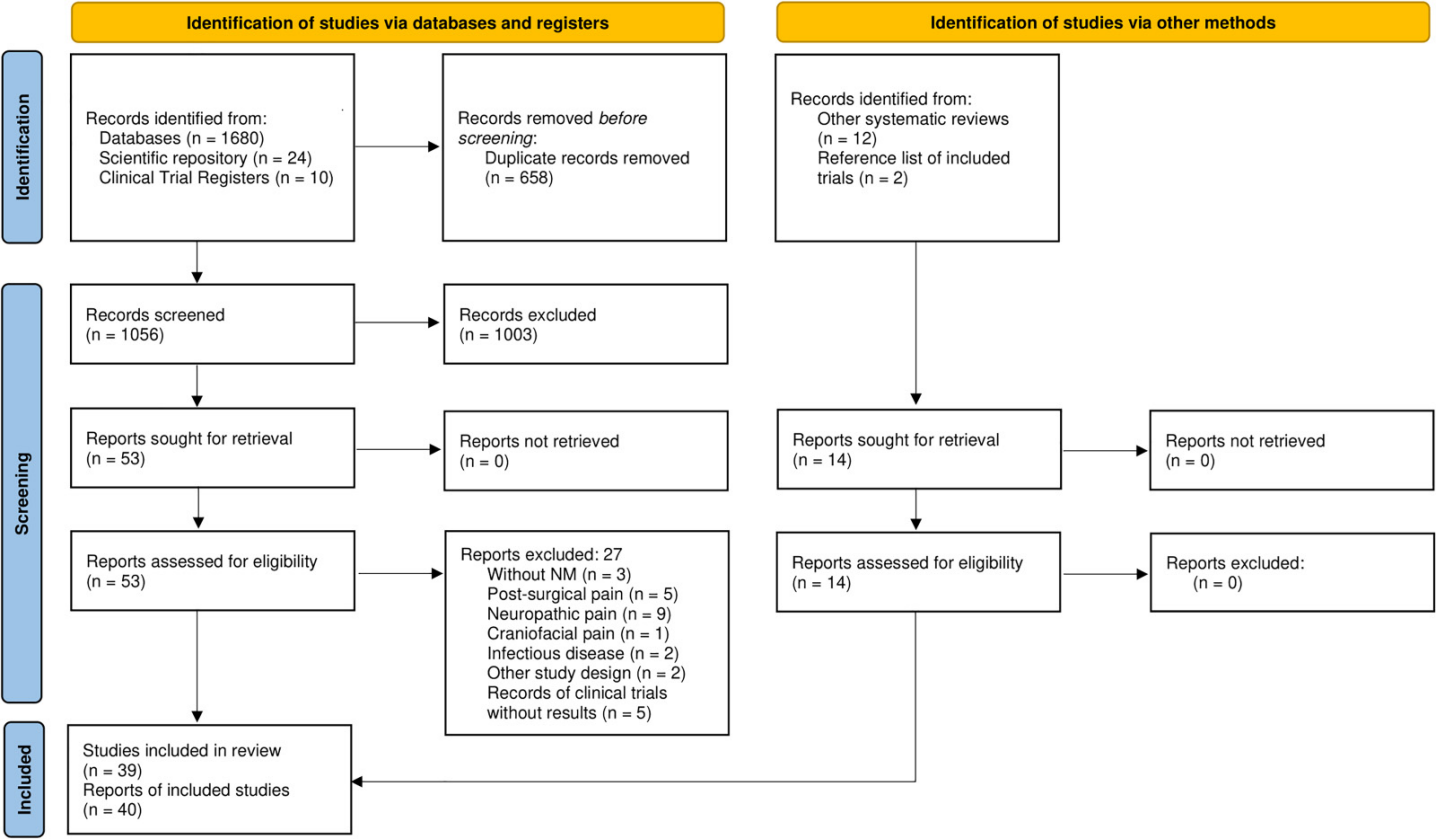
PRISMA flow diagram.

A total of 1714 records were identified, of which 658 were duplicates. Thus, 1056 were screened by title and abstract and 53 followed for full-text screening. Of these, 27 reports were excluded and 40 remained for this systematic review, considering that 14 studies were included from other sources. Taking into account that two reports were related to the same study,^49,50^ a total of 39 studies were included^49–88^ (Figure 1). The excluded reports are listed in Supplemental File 4 with the reasons for exclusion.

Included studies (n = 39) cover 10 musculoskeletal conditions: hand osteoarthritis (n = 3),^49,50,80,87^ lateral epicondylitis (n = 4),^61,83,84,86^ ankle sprain (n = 1),^72^ shoulder impingement syndrome (n = 1),^85^ rheumatoid arthritis (n = 2),^78,79^ unspecified musculoskeletal pain (n = 1),^77^ fibromyalgia (n = 1),^76^ plantar heel pain syndrome (n = 1),^75^ low back pain (n = 17),^58–60,62–71,73,74,81,82^ and neck pain (n = 8).^51–57,88^

Studies characteristics are summarised in Tables 2 to 11. The characteristics of the interventions are presented in Supplemental File 5.

**Table 2.** Characteristics of individual studies (Low back pain).

Study ID		Characteristics of participants				Interventions		Outcome timing	Outcomes measured
Authors (Year)	Study design	Sample size (n)	MSK condition	Mean age (SD)	Female n (%)	NM group	Comparison group		
Sousa Filho et al. (2022)	RCT	Total (31) EG (17) CG (14)	Chronic LBLP	Total: not informed EG: 42.5 (±13.1) CG: 34.5 (±14.8)	Total: 22 (70.96%) EG: 12 (70.6%) CG: 10 (71.4%)	**Multimodal intervention (3-week program / 7 sessions):** 1) NM exercises - Sliding / tensioning techniques combined - Active technique 2) Extension exercises	**Single intervention (3-week program / 7 sessions):** 1) Extension exercises	Post-intervention: 3 weeks Follow-up: 1-month post-intervention	- Pain intensity (NPRS) - Functional status (RMDQ)
Adnan et al. (2022)	RCT	Total (32) EG (16) CG(16)	Low back pain that radiates up to the knee	Total: 38.81 (±9.94) EG: not informed CG: not informed	Total: 22 (68.75%) EG: not informed CG: not informed	**Multimodal intervention** **(4-week program / 20 sessions):** 1) NM - Tensioning technique - Passive technique 2) Transcutaneous electrical nerve stimulation (TENS) therapy	**Multimodal intervention (4-week program / 20 sessions):** 1) Mulligan bent leg raise technique 2) Transcutaneous electrical nerve stimulation (TENS) therapy	Post-intervention: 4 weeks	- Pain intensity (NPRS) - Functional status (ODI) - ROM in hip flexion with extended knee (SLR Test)
González et al. (2021)	RCT	Total (51) EG (26) CG (25)	Subacute non-specific LBP	Total: not informed EG: 42.73 (±13.64) CG: 44.64 (±11.28)	Total: 31 (60.8%) EG: 13 (50%) CG: 18 (72%)	**Single intervention** **(1 session):** 1) NM - Sliding technique - Passive / Active techniques combined	**Single intervention** **(1 session):** 1) Sham NM	Post-intervention: 1 session Follow-up: 1 week after intervention	- Pain intensity (VAS) - ROM in hip flexion with extended knee (SLR Test)
Patel et al. (2020)	RCT	Total (52) EG (26) CG (26)	Chronic LBP (> 6 months)	Total: 25–40 (range) EG: not informed CG: not informed	Total: not informed EG: not informed CG: not informed	**Single intervention** **(2-week program / 10 sessions):** 1) NM - Techniques used not reported	**Single intervention (2-week program / 10 sessions):** 1) Muscle energy technique	Post-intervention: 2 weeks	- Pain intensity (VAS) - Functional status (ODI) - ROM in hip flexion with extended knee (SLR Test)
Kurt et al. (2020)	RCT	**Baseline:** Total (60) EG (30) CG (30) **T1:** Total (41) EG (20) CG (21)	Chronic / Acute LBP	Total: not informed EG: 39.45 (±8.55) CG: 38.33 (±9.70)	Total: 19 (46%) EG: 9 (45%) CG: 10 (47%)	**Multimodal intervention (3-week program / 15 sessions):** 1) Local hot pack 2) Electrotherapy - TENS—Ultrasound 3) NM exercise in a slump position - Sliding technique - Active technique	**Multimodal intervention (3-week program / 15 sessions):** 1) Local hot pack 2) Electrotherapy - TENS - Ultrasound	Post-intervention: 3 weeks	- Pain intensity (VAS) - Functional status (ODI) - ROM in hip flexion with extended knee (SLR Test) - Static balance (baropedographic) - Gait parameters (baropedographic)
Moksha et al. (2019)	RCT	Total (60) EG 1 (30) EG 2 (30)	Non-specific LBP with unilateral lower limb symptoms	Total: 25–60 (range) EG 1: not informed EG 2: not informed	Total: 44 (73.33%) EG 1: 21 (70%) EG 2: 23 (76.66%)	**NM group 1:** **Multimodal intervention (5 sessions):** 1) Slider NM technique - Passive / Active techniques combined 2) Hot packs 3) Home exercise program **NM group 2:** **Multimodal intervention (5 sessions):** 1) Tensioner NM technique - Passive / Active techniques combined 2) Hot packs 3) Home exercise program	NA	Post-intervention: 5 sessions	- Pain intensity (NPRS) - Functional status (MODI) - ROM in hip flexion with extended knee (SLR Test)
Kirthika et al. (2016)	RCT	Total (60) EG (30) CG (30)	Subacute non-radicular LBP	Total: not informed EG: not informed CG: not informed	Total: not informed EG: not informed CG: not informed	**Multimodal intervention (6-week program):** 1) Postural advice 2) Stretching exercises for the lower limbs 3) Progressive core stabilisation exercise protocol 4) Endurance exercise protocol for trunk extensor muscles 5) NM (slump stretching) - Sliding technique - Passive / Active techniques combined	**Multimodal intervention (6-week program):** 1) Postural advice 2) Stretching exercises for the lower limbs 3) Progressive core stabilisation exercise protocol 4) Endurance exercise protocol for trunk extensor muscles	Post-intervention: 6 weeks	- Pain intensity (VAS) - Functional status (MODI)
Jaidka et al. (2016)	qRCT	Total (45) EG (15) CG 1 (15) CG 2 (15)	Chronic LBP	Total: 25–50 (range) EG: 35.73 (±9.30) CG 1: 39.73 (±10) CG 2: 38.80 (±8.04)	Total: not informed EG: not informed CG 1: not informed CG 2: not informed	**Multimodal intervention** **(6 sessions):** 1) NM (slump stretching) - Tensioning technique - Passive / Active techniques combined 2) Shor wave diathermy 3) Lumbar stabilisation exercises 4) TENS	**Multimodal intervention** **(6 sessions):** **CG 1:** 1) Posteroanterior spinal mobilisation 2) Short wave diathermy 3) Lumbar stabilisation exercises 4) TENS **CG 2:** 1) Short wave diathermy 2) Lumbar stabilisation exercises 3) TENS	Post-intervention: 6 sessions	- Pain intensity (VAS) - Functional status (ODI) - ROM in hip flexion with extended knee (SLR Test)
Mansuri & Shah (2015)	qRCT	Total (60) EG (30) CG (30)	Non-radicular LBP	Total: 25–50 (range) EG: not informed CG: not informed	Total: not informed EG: not informed CG: not informed	**Multimodal intervention** **(6 sessions):** 1) Hot packs 2) Isometric exercises 3) NM (slump stretching) - Tensioning technique - Passive / Active techniques combined	**Multimodal intervention** **(6 sessions):** 1) Hot packs 2) Isometric exercises	Post-intervention: 6 sessions	- Pain intensity (NPRS) - Functional status (MODI) - ROM of active knee extension in the position of hip flexion (AKE Test)
Karthikeyan et al. (2014)	qRCT	Total (40) EG (20) CG (20)	Non-radicular LBP	Total: 20–45 (range) EG: not informed CG: not informed	Total: 17 (42.5%) EG: 7 (35%) CG: 10 (50%)	**Multimodal intervention** **(2-week program / 12 sessions):** 1) Warm up exercises 2) NM (slump stretching) - Tensioning technique - Passive / Active techniques combined 3) Static spinal exercise 3) Warm down exercise	**Multimodal intervention** **(2-week program / 12 sessions):** 1) Warm up exercises 2) Mobilisation (Grade III, IV) 3) Static spinal exercise 3) Warm down exercise	Post-intervention: 2 weeks	- Pain intensity (NPRS) - Functional status (ODI)
Ravinder et al. (2014)	RCT	Total (40) EG (20) CG (20)	Non-radicular LBP	Total: 18–70 (range) EG: not informed CG: not informed	Total: 12 (30%) EG: 6 (30%) CG: 6 (30%)	**Multimodal intervention** **(3-week program / 6 sessions):** 1) NM (slump stretching) - Tensioning technique - Passive / Active techniques combined 2) Exercises	**Multimodal intervention** **(3-week program / 6 sessions):** 1) Cognitive intervention (ergonomic advice) 2) Exercises	Post-intervention: 3 weeks	- Pain intensity (NPRS) - Functional status (MODI)
Patel et al. (2014)	RCT	Total (50) EG (25) CG (25)	LBP	Total: 30–60 (range) EG: not informed CG: not informed	Total: not informed EG: not informed CG: not informed	**Single intervention** **(4-week program / 16 sessions):** 1) NM (slump stretching) - Tensioning technique - Passive / Active techniques combined	**Single intervention** **4-week program / 16 sessions):** 1) Mulligan bent leg raising	Post-intervention: 4 weeks	- Pain intensity (VAS) - ROM in hip flexion with extended knee (SLR Test)
Jain et al. (2012)	RCT	Total (30) EG (not informed) CG (not informed)	Subacute non-radicular LBP	Total: not informed EG: 34.26 (±5.66) CG: 33 (±6.85)	Total: 19 (63%) EG: not informed CG: not informed	**Multimodal intervention** **(4-week program / 9 sessions):** 1) Posteroanterior mobilisation 2) Therapeutic exercises 3) NM (slump stretching) - Tensioning technique - Passive / Active techniques combined	**Multimodal intervention (4-week program / 18 sessions):** 1) Posteroanterior mobilisation 2) Therapeutic exercises	Post-intervention: 4 weeks Follow-up: 1 week after intervention	- Pain intensity (VAS) - Functional status (MODI)
Malik et al. (2012)	RCT	Total (40) EG 1 (15) EG 2 (13) CG (12)	LBP	Total: 18–60 (range) EG 1: not informed EG 2: not informed CG: not informed	Total: not informed EG 1: not informed EG 2: not informed CG: not informed	**NM group 1:** **Multimodal intervention** **(3-week program / 6 sessions):** 1) Advice 2) Lumbar stabilisation exercises 3) NM (straight leg raise stretching) - Tensioning technique - Passive technique **NM group 2:** **Multimodal intervention** **(3-week program / 6 sessions):** 1) Advice 2) Lumbar stabilisation exercises 3) NM (slump stretching) - Tensioning technique - Passive / Active techniques combined	**Multimodal intervention** **(3-week program / 15 sessions):** 1) Advice 2) Lumbar stabilisation exercises	Post-intervention: 3 weeks	- Pain intensity (VAS) - ROM in hip flexion with extended knee (SLR Test)
Nagrale et al. (2012)	RCT	Total (60) EG (30) CG (30)	Chronic non-radicular LBP	Total: 18–60 (range) EG: 38.2 (±3.47) CG: 37.76 (±4.70)	Total: 39 (65%) EG: 21 (70%) CG: 18 (60%)	**Multimodal intervention** **(3-week program / 6 sessions):** 1) Stationary bicycle warm-up 2) Lumbar spine mobilisation 3) Lumbar stabilisation exercises 4) NM (slump stretching) - Tensioning technique - Passive / Active techniques combined	**Multimodal intervention** **(3-week program / 6 sessions):** 1) Stationary bicycle warm-up 2) Lumbar spine mobilisation 3) Lumbar stabilisation exercises	Post-intervention: 3 weeks Follow-up: 3 weeks after intervention	- Pain intensity (NPRS) - Functional status (MODI)
Machado & Bigolin (2010)	RCT	Total (9) EG (5) CG (4)	Chronic LBP	Total: 44.22 (±8.54) EG: not informed CG: not informed	Total: 7 (77.77%) EG: not informed CG: not informed	**Single intervention** **(20 sessions):** 1) NM exercises - Techniques used not reported	**Single intervention** **(20 sessions):** 1) Stretching exercises	Post-intervention: 20 sessions	- Pain intensity (VAS) - Functional status (RMDQ) - Flexibility (Knee flexion ROM) - Flexibility (finger-to-ground distance)
Cleland et al. (2006)	RCT	Total (30) EG (16) CG (14)	Non-radicular LBP	Total: 38.7 (±11.6) EG: 39.4 (±11.3) CG: 40.0 (±12.2)	Total: 21 (70%) EG: 11 (68.75%) CG: 10 (71.42%)	**Multimodal intervention** **(3-week program / 6 sessions):** 1) Stationary bicycle warm-up 2) Lumbar spine mobilisation 3) Lumbar stabilisation exercises 4) NM (slump stretching) - Tensioning technique - Passive / Active techniques combined	**Multimodal intervention** **(3-week program / 6 sessions):** 1) Stationary bicycle warm-up 2) Lumbar spine mobilisation 3) Lumbar stabilisation exercises	Post-intervention: 3 weeks	- Pain intensity (NPRS) - Functional status (MODI)

Abbreviations: EG – experimental group; CG – comparison group; LBP – low back pain; LBLP – low back-related leg pain; MSK – musculoskeletal; IQR – interquartile range; NM - Neural Mobilisation; VAS – Visual Analogue Scale; ODI – Oswestry Disability Index; MODI – Modified Oswestry Disability Index; SLR – Straight Leg Raise; NPRS – Numeric Pain Rating Scale; AKE – Active Knee Extension; TENS – Transcutaneous electrical nerve stimulation; RMDQ – Roland Morris Disability Questionnaire; ROM - Range of Motion; RCT - Randomised Controlled Trial; qRCT - quasi Randomised Trial.

**Table 3.** Characteristics of individual studies (Neck pain).

Study ID		Characteristics of participants				Interventions		Outcome timing	Outcomes measured
Authors (Year)	Study design	Sample size (n)	MSK condition	Mean age (SD) or Median (IQR)	Female n (%)	NM group	Comparison group		
**Cabrera-Martos et al. (2020)**	RCT	Total (40) EG (20) CG (20)	Chronic neck pain	Total: not informed EG: 28.84 (±5.78) CG: 32.5 (±4.68)	Total: 30 (75%) EG: 14 (70%) CG: 16 (80%)	**Multimodal intervention** **(4-week program / 12 sessions):** 1) Myofascial release (in the first 2 weeks) 2) NM (in the last 2 weeks) - Sliding technique - Active technique	**Minimal intervention** **(4 week-program):** 1) A booklet with information on neck pain		- Pain (BPI) - Pain intensity (VAS) - Functional status (NOOS) - The percentage of active trigger points
**Fernández-Carnero et al. (2019)**	RCT	Total (54) EG (27) CG (27)	Chronic neck pain	Total: 20.91 (±2.64) EG: 21.83 (±3.16) CG: 19.93 (±1.43)	Total: 41 (75.92%) EG: 23 (85.2%) CG: 18 (66.7%)	**Single intervention** **(1 session):** 1) NM - Tensioning technique - Passive technique	**Single intervention** **(1 session):** 1) Sham NM	Post-intervention: 1 session	- Pain intensity (VAS) - Cervical active ROM (CROM device) - Conditioned Pain Modulation (tourniquet test & a digital algometer)
**Masullo (2018)**	RCT	Total (11) EG (5) CG (6)	Non-specific mechanical neck pain without radiation on arms	Total: 40.73 (?) EG: 49.6 (?) CG: 36.3 (?)	Total: 8 (72.72%) EG: 4 (80%) CG: 4 (66.66%)	**Multimodal intervention** **(2 sessions):** 1) Osteopathic manipulative techniques 2) NM (ULTT 1a) - Tensioning technique - Passive technique	**Multimodal intervention** **(2 sessions):** 1) Osteopathic manipulative techniques 2) Articulations of the acromioclavicular joint	Post-intervention: 2 sessions	- Pain intensity (VAS) - Functional status (NDI) - PPT over the cervical spine (algometer)
**Khan et al. (2015)**	RCT	Total (40) EG (20) CG (20)	Cervicobrachial Pain Syndrome (subacute / chronic)	Total: 30–60 (range) EG: 42.70 (±9.95) CG: 52.60 (±6.15)	Total: not informed EG: not informed CG: not informed	**Single intervention** **(6-week program / (?) sessions)** 1) NM - Sliding technique - (?)	**Single intervention** **(6-week program / (?) sessions)** 1) Cervical spine mobilisation	Post-intervention: 6 weeks	- Pain intensity (VAS) - Functional status (NDI) - Cervical active ROM (inclinometer)
**Gupta et al. (2012)**	RCT	Total (34) EG (16) CG (18)	Cervicobrachial Pain Syndrome (subacute)	Total: 18–40 (range) EG: 29 (?) median CG: 29.5 (?) median	Total: 16 (47%) EG: 6 (37.5%) CG: 10 (55.55%)	**Single intervention** **(1-week program / 5 sessions):** 1) NM (Median nerve) - Sliding technique - Passive / Active techniques combined	**Single intervention** **(1-week program / 5 sessions):** 1) Cervical spine mobilisation	Post-intervention: 1 week	- Pain intensity (VAS) - Functional status (NDI & CBSQ) - Passive elbow extension ROM in the ULNT 1 position (inclinometer)
**Marks et al. (2011)**	RCT	Total (20) EG (10) CG (10)	Cervicobrachial Pain Syndrome	Total: not informed EG: 52.6 (±12.5) CG: 53.7 (±9.0)	Total: 16 (80%) EG: 8 (80%) CG: 8 (80%)	**Single intervention** **(1 session):** 1) NM - Sliding technique - (?)	**Single intervention** **(1 session):** 1) Cervical spine mobilisation	Post-intervention: 1 session Follow-up: 1 week after intervention	- Pain intensity (VAS) - Arm pain intensity (VAS) - Cervical active ROM (CROM device) - Passive elbow extension ROM in the ULNT 1 position (Mathcad 13 software [Mathsoft Engineering & Education Inc. 2005])
**Chhabra et al. (2008)**	RCT	Total (37) EG (19) CG (18)	Cervicobrachial Pain Syndrome (subacute)	Total: not informed EG: not informed CG: not informed	Total: not informed EG: not informed CG: not informed	**Multimodal intervention** **(6-week program / up to 7 sessions):** 1) Hot pack 2) NM - Tensioning technique - Passive technique 3) Home exercises	**Multimodal intervention** **(6-week program / up to 7 sessions):** 1) Hot pack 2) Cervical lateral glide 3) Home exercises	Post-intervention: 6 weeks	- Pain intensity (VAS) - Functional status (NDI) - Passive elbow extension ROM in the ULNT 1 position (universal goniometer)
**Allison et al. (2002)**	Cross over trial	**Baseline:** Total (30) EG 1 (10) EG 2 (10) CG (10) **T1:** Total (30) EG 1 (20) EG 2 (10) CG (10)	Cervicobrachial Pain Syndrome (chronic)	Total: 54 (±13.5) EG 1: 50 (±19.5) EG 2: 61 (±8.5) CG: 52.5 (±10)	Total: 20 (66.6%) EG 1: 6 (60%) EG 2: 8 (80%) CG: 6 (60%)	**Multimodal intervention** **(8-week program / (?) sessions):** NEURAL TREATMENT (direct approach to the neural tissue) 1) Cervical spine mobilisation (cervical lateral glide) 2) Shoulder girdle oscillation (caudal cephalic mobilisation) 3) Muscle re-education 4) Home exercise program	**CG 1:** **Multimodal intervention** **(8-week program / (?) sessions):** ARTICULAR TREATMENT (indirect or non-specific intervention) 1) Glenohumeral mobilisation 2) Thoracic mobilisation 3) Home exercise program **CG 2:** **No intervention** **(8-week program):** The Comparison group received no intervention for 8 weeks, so they were allocated to the specific neural treatment (NT) group as a crossover protocol.	Post-intervention: 8 weeks	- Pain intensity (VAS) - Pain quality (SF-MGP) - Functional status (NPQ)

Abbreviations: EG – experimental group; CG – comparison group; MSK – musculoskeletal; IQR – interquartile range; NM - Neural Mobilisation; BPI – Brief Pain Inventory; VAS – Visual Analogue Scale; NOOS – Neck Outcome Score; ROM - Range of Motion; CROM – Cervical Range of Motion; NDI – Neck Disability Index; CBSQ – Cervicobrachial Symptom Questionnaire; SF-MGP – Short-form McGill Pain Questionnaire; NPQ – Northwick Park Neck Pain Questionnaire; PPT - Pressure Pain Threshold; ULTT - Upper Limb Tension Test; ULNT - Upper Limb Nerve Test; RCT - Randomised Controlled Trial.

**Table 4.** Characteristics of individual studies (Hand osteoarthritis).

Study ID		Characteristics of participants				Interventions		Outcome timing	Outcomes measured
Authors (Year)	Study design	Sample size (n)	MSK condition	Mean age (SD) or Median (IQR)	Female n (%)	NM group	Comparison group		
**Pedersini et al. (2021)**	RCT	Total (72) EG (36) CG (36)	Hand osteoarthritis (severity of 3 to 4 on the Kallgren / Lawrence scale)	Total: 71 (±11) EG: 71 (±11) CG: 69 (±12)	Total: 40 (55%) EG: 21 (58%) CG: 19 (52%)	**Multimodal intervention** **(4-week program / 12 sessions):** 1) NM - Sliding technique - Passive technique 2) Hand stability exercises	**Multimodal intervention** **(4-week program / 12 sessions):** 1) Robotic-assisted passive mobilisation 2) Hand stability exercises	Post-intervention: 4 weeks Follow-up: 3 months after intervention	- Pain intensity (VAS) a) key pinch b) over the last 24 h c) over the last week - PPT over the 1^st^ CMC joint (algometer) - Grip strength (dynamometer) - Pinch strength (mechanical pinch gauge)
**Villafañe et al. (2013b)**	RCT	Total (60) EG (30) CG (30)	Hand osteoarthritis (stage III or IV secondary CMC joint according to the Eaton-Littler-Burton classification system based on radiographic findings)	Total: 82 (±6) EG: 82 (±2) CG: 83 (±1)	Total: 51 (85%) EG: 27 (90%) CG: 24 (80%)	**Multimodal intervention** **(4-week program / 12 sessions):** 1) NM - Sliding technique - Passive technique 2) Joint mobilisation 3) Hand stability exercises	**Single intervention** **(4-week program / 12 sessions):** 1) Sham intervention (detuned ultrasound therapy)	Post-intervention: 4 weeks Follow-up 1: 1 month after intervention Follow-up 2: 2 months after intervention	- Pain intensity (VAS) a) key pinch - PPT over the 1^st^ CMC joint (algometer) - Grip strength (dynamometer) - Pinch strength (mechanical pinch gauge)
**Villafañe et al. (2012/2013a)**	RCT	Total (60) EG (30) CG (30)	Hand osteoarthritis (stage III or IV secondary CMC joint according to the Eaton-Littler-Burton classification system based on radiographic findings)	Total: 70–90 (range) EG: 80.97 (±2.93) CG: 81.73 (±2.93)	Total: 54 (90%) EG: 28 (92.86%) CG: 26 (84.62%)	**Single intervention** **(4-week program / 6 sessions):** 1) NM - Sliding technique - Passive technique	**Single intervention** **(4-week program / 6 sessions):** 1) Placebo intervention (intermittent ultrasound therapy)	Post-intervention: 4 weeks Follow-up 1: 1 month after intervention Follow-up 2: 2 months after intervention	- PPT over the 1^st^ CMC joint - Tripod pinch strength (mechanical pinch gauge) - Tip pinch strength (mechanical pinch gauge)

Abbreviations: EG – experimental group; CG – comparison group; MSK – musculoskeletal; IQR – interquartile range; NM - Neural Mobilisation; CMC – carpometacarpal; VAS – Visual Analogue Scale; PPT - Pressure Pain Threshold; RCT - Randomised Controlled Trial.

**Table 5.** Characteristics of individual studies (Lateral epicondylitis).

Study ID		Characteristics of participants				Interventions		Outcome timing	Outcomes measured
Authors (Year)	Study design	Sample size (n)	MSK condition	Mean age (SD) or Median (IQR)	Female n (%)	NM group	Comparison group		
**Yilmaz et al. (2022)**	RCT	**Baseline:** Total (40) EG (20) CG (20) **T1 and T2:** Total (35) EG (17) CG (18) **T3 (follow-up):** Total (34) EG (16) CG (18)	Chronic lateral epicondylitis	Total: 42.8 (±8.9) EG: 42.7 (±7.57) CG: 42.9 (±10.2)	Total: 26 (65%) EG: 14 (70%) CG: 12 (60%)	**Multimodal intervention** **(6-week program / 9 face-to-face sessions and ≈ 42 home sessions):** 1) NM - Tensioning technique - Passive / Active techniques combined 2) Home program (patient education and eccentric exercises)	**Single intervention** **(6-week program / ≈ 42 home sessions):** 1) Home program (patient education and eccentric exercises)	Post-intervention: 6 weeks Follow-up: 6 weeks after intervention	- Pain intensity (VAS) a) rest pain level b) night pain level c) activity pain level - Functional status of the upper extremity (DASH questionnaire) - Grip strength (dynamometer) a) painless grip strength when elbow in flexion b) maximum grip strength when elbow in flexion c) painless grip strength when elbow in extension d) maximum grip strength when elbow in flexion - Pinch strength (mechanical pinch gauge) a) key pinch grip strength b) tip pinch grip strength - Wrist active ROM (goniometer) a) extension b) flexion c) radial deviation d) ulnar deviation
**Dabholkar et al. (2013)**	qRCT	Total (40) EG (20) CG (20)	Lateral epicondylitis	Not informed	Not informed	**Multimodal intervention** **(4-week program / 16 face-to-face sessions and ≈ 28 home sessions):** 1) NM - Sliding / Tensioning techniques combined - Passive / Active techniques combined 2) MWM (head of the radius) 3) Exercise program	**Single intervention** **(4-week program / 16 sessions):** 1) Exercise program	Post-intervention: 4 weeks	- Pain intensity (VAS) - Functional status (Patient Rated Tennis Elbow Evaluation Questionnaire) - Painless grip strength when elbow in flexion (dynamometer) - PPT over the epicondyle (algometer)
**Drechsler et al. (1997)**	RCT	Total (18) EG (8) CG (10)	Chronic lateral epicondylitis (tennis elbow)	Total: 46 (±?) EG: 46.4 (±?) CG: 45.5 (±?)	Total: 10 (55%) EG: 4 (50%) CG: 6 (60%)	**Multimodal intervention** **(6 to 8-week program / ≈ 12 to 16 sessions):** 1) NM - Tensioning technique - Passive / Active techniques combined 2) Joint mobilisation (head of the radius)	**Multimodal intervention** **(6 to 8-week program / ≈ 12 to 16 sessions):** 1) Ultrasound 2) Transverse friction massage 3) Exercises (stretching and strengthening exercises for the extensors of the wrist)	Post-intervention: 6 to 8 weeks Follow-up: 3 months after intervention	**CONTINUOUS VARIABLES:** - Functional status (self-report questionnaire) - Maximum grip strength when elbow in flexion - ULNT 2b ROM (goniometer) **CATEGORICAL VARIABLES:** - 3^rd^ finger extension test (painful vs. nonpainful) - Elbow extension ROM (limited or not limited) compared to the uninvolved elbow - Radial head mobility (hypomobile, normal, or hypermobile) compared to the contralateral side
**Vicenzino et al. (1996)**	Crossover trial	Total (15) Subjects received the three conditions in a randomised sequence.	Lateral epicondylitis	Total: 22.5–62 (range)	Total: 8 (53%)	**Single intervention** **(1 session):** 1) Cervical contralateral glide with the affected upper limb maintained in a ULTT 2b position	**CG 1:** **Single intervention** **(1 session):** 1) Placebo **CG 2:** **No intervention**	Post-intervention: 3 days	- Pain intensity (VAS) a) rest pain level b) over the previous 24h - Functional status over the previous 24 h (VAS) - PPT (algometer) - Painless grip strength when elbow in extension (dynamometer) - ULNT 2b ROM (electro goniometer)

Abbreviations: EG – experimental group; CG – comparison group; MSK – musculoskeletal; IQR – interquartile range; NM - Neural Mobilisation; VAS – Visual Analogue Scale; DASH – The Disabilities of the Arm, Shoulder, and Hand; MWM – mobilisation with movement; PPT - Pressure Pain Threshold; ROM - Range of Motion; ULNT - Upper Limb Nerve Test; ULTT - Upper Limb Tension Test; RCT - Randomised Controlled Trial; qRCT - quasi Randomised Trial.

**Table 6.** Characteristics of individual studies (Rheumatoid arthritis).

Study ID		Characteristics of participants				Interventions		Outcome timing	Outcomes measured
Authors (Year)	Study design	Sample size (n)	MSK condition	Mean age (SD) or Median (IQR)	Female n (%)	NM group	Comparison group		
**Lau et al. (2019)**	RCT	Total (21) EG (11) CG (10)	Rheumatoid arthritis	Total: 57.5 (±7.1) EG: (?) CG: (?)	Total: 21 (100%) EG: 11 (100%) CG: 10 (100%)	**Single intervention** **(4 to 8-week program / ≈ 28 to 56 sessions):** 1) NM - Tensioning technique - Active technique	**Single intervention** **(4 to 8-week program / ≈ 28 to 56 sessions):** 1) Joint mobilisation exercises	Post-intervention: 4 to 8 weeks	- Impact of disease on patient´s daily lives (RAID – pain intensity, functional status, fatigue, sleep, physical well-being, emotional well-being, coping) - Erythrocyte sedimentation rate
**Lo et al. (2017)**	RCT	Total (9) EG (5) CG (4)	Rheumatoid arthritis	Total: 57.5 (±8.8) EG: 55.2 (±11.4) CG: 59.8 (±6.3)	Total: 8 (89%) EG: 5 (100%) CG: 3 (75%)	**Single intervention** **(4-week program / ≈ 28 sessions)** 1) NM - Sliding / Tensioning techniques - Active technique	**Single intervention** **(4-week program / ≈ 28 sessions)** 1) Joint mobilisation exercises	Post-intervention: 4 weeks	- Pain intensity (NRS) - Severity of pain and inflammation (RAPS) - C-reactive protein - Erythrocyte sedimentation rate

Abbreviations: EG – experimental group; CG – comparison group; MSK – musculoskeletal; IQR – interquartile range; NM - Neural Mobilisation; RAID – The Rheumatoid Arthritis Impact of Disease; NRS – Numeric Pain Rating Scale; RAPS – Rheumatoid Arthritis Pain Scale; RCT - Randomised Controlled Trial.

**Table 7.** Characteristics of individual studies (Ankle sprain).

Study ID		Characteristics of participants				Interventions		Outcome timing	Outcomes measured
Authors (Year)	Study design	Sample size (n)	MSK condition	Mean age (SD) or Median (IQR)	Female n (%)	NM group	Comparison group		
**Plaza-Manzano et al. (2016)**	RCT	Total (56) EG (28) CG (28)	Recurrent plantar flexion with inversion (PFI) ankle sprains	Total: 24.3 (±2.5) EG: 24.4 (±2.4) CG: 24.1 (±2.4)	Total: 17 (30%) EG: 9 (32%) CG: 8 (28%)	**Multimodal intervention** **(4-week program / 8 sessions):** 1) NM - Tensioning technique - Passive technique 2) Proprioceptive / Strengthening exercises (eccentric exercises) 3) Joint mobilisation (talocrural and distal tibiofibular joints)	**Single intervention** **(4-week program / 8 sessions):** 1) Proprioceptive / Strengthening exercises (eccentric exercises)	Post-intervention: 4 weeks Follow-up: 1 month after intervention	- Pain intensity (VAS) - Functional ankle instability (CAIT) - PPT (algometer) a) the anterior talofibular ligament b) the calcaneofibular ligament c) tibial malleolus d) fibular malleolus - Muscle strength (dynamic dynamometer) a) Flexion b) Extension - Active ROM (goniometer) a) flexion b) extension

Abbreviations: EG – experimental group; CG – comparison group; MSK – musculoskeletal; IQR – interquartile range; VAS – Visual Analogue Scale; CAIT – The Cumberland Ankle Instability Tool; ROM - Range of Motion; RCT - Randomised Controlled Trial.

**Table 8.** Characteristics of individual studies (Shoulder impingement syndrome).

Study ID		Characteristics of participants				Interventions		Outcome timing	Outcomes measured
Authors (Year)	Study design	Sample size (n)	MSK condition	Mean age (SD) or Median (IQR)	Female n (%)	NM group	Comparison group		
Akhtar et al. (2020)	RCT	Total (80) EG (40) CG (40)	Shoulder impingement syndrome	Total: (?) EG: 36.38 (±8.93) CG: 34.40 (±9.32)	Total: 58 (72.5%) EG: 32 (80%) CG: 26 (65%)	**Multimodal intervention** **(11-week program / 33 sessions):** 1) NM - Sliding / Tensioning techniques - Passive / Active techniques combined 2) Pulsed Short Wave Diathermy (SWD) 3) Transcutaneous Electrical Nerve Stimulator (TENS) 4) Exercises (strengthening and stretching exercises)	**Single intervention** **(11-week program / 33 sessions):** 1) Pulsed Short Wave Diathermy (SWD) 2) Ultrasonic therapy 3) Transcutaneous Electrical Nerve Stimulator (TENS) 4) Exercises (strengthening and stretching exercises)	Post-intervention: T1: 5 weeks T2: 11 weeks	- Pain intensity (VAS) - Functional disability (UCLA score)

Abbreviations: EG – experimental group; CG – comparison group; MSK – musculoskeletal; IQR – interquartile range; NM - Neural Mobilisation; VAS – Visual Analogue Scale; UCLA – The University of California at Los Angeles; RCT - Randomised Controlled Trial.

**Table 9.** Characteristics of individual studies (Plantar heel pain syndrome).

Study ID		Characteristics of participants				Interventions		Outcome timing	Outcomes measured
Authors (Year)	Study design	Sample size (n)	MSK condition	Mean age (SD) or Median (IQR)	Female n (%)	NM group	Comparison group		
**Saban et al. (2014)**	RCT	Total (69) EG (36) CG (33)	Plantar heel pain syndrome	Total: 53 (±13) EG: 54 (±12) CG: 52 (±13)	Total: 39 (57%) EG: 17 (47%) CG: 22 (67%)	**Multimodal intervention** **(4 to 6-week program / ≈ 8 sessions):** 1) NM - Tensioning technique - Active technique 2) Deep massage therapy 3) Self-stretch exercises	**Multimodal intervention** **(4 to 6-week program / ≈ 8 sessions):** 1) Ultrasound 2) Self-stretch exercises	Post-intervention: 4 to 6 weeks	- Pain intensity (VAS) - Functional status (CAT)

Abbreviations: EG – experimental group; CG – comparison group; MSK – musculoskeletal; IQR – interquartile range; NM - Neural Mobilisation; VAS – Visual Analogue Scale; CAT – Foot & Ankle Computerized Adaptive Test; RCT - Randomised Controlled Trial.

**Table 10.** Characteristics of individual studies (Unspecified musculoskeletal pain).

Study ID		Characteristics of participants				Interventions		Outcome timing	Outcomes measured
Authors (Year)	Study design	Sample size (n)	MSK condition	Mean age (SD) or Median (IQR)	Female n (%)	NM group	Comparison group		
**Mateus et al. (2020)**	RCT	Total (26) EG (13) CG (13)	Unspecified MSK pain in institutionalised older adults	Total: (?) EG: 82 (±12.5) CG: 84 (±9.22)	Total: 20 (77%) EG: 11 (84.6%) CG: 9 (69.2%)	**Multimodal intervention** **(8-week program / 16 sessions):** 1) NM - Sliding technique - Active technique 2) Therapeutic exercise	**Single intervention** **(8-week program / 16 sessions):** 1) Therapeutic exercise	Post-intervention: 8 weeks	- Pain intensity in the last week (NPRS) - Flexibility (SLR Test) - Static balance (Tandem Stance Test) - Gait velocity (4-m Gait Speed Test) - Mobility (Timed Up and Go Test)

Abbreviations: EG – experimental group; CG – comparison group; MSK – musculoskeletal; IQR – interquartile range; NPRS – Numeric Pain Rating Scale; SLR – Straight Leg Raise.

**Table 11.** Characteristics of individual studies (Fibromyalgia).

Study ID		Characteristics of participants				Interventions		Outcome timing	Outcomes measured
Authors (Year)	Study design	Sample size (n)	MSK condition	Mean age (SD) or Median (IQR)	Female n (%)	NM group	Comparison group		
**Torres et al. (2015)**	RCT	Total (48) EG (24) CG (24)	Fibromyalgia	Total: (?) EG: 53 (±10.2) CG: 53 (±7.6)	Total: 39 (81.25%) EG: 19 (79%) CG: 20 (83%)	**Single intervention** **(8-week program / 16 sessions):** 1) NM - Sliding / Tensioning techniques - Active technique	1) Usual activities 2) Advice	Post-intervention: 8 weeks	- ROM of neurodynamic movements [ULNT 1, ULNT 2a, ULNT 2b, ULNT 3, Slump Test, SLR Test] (goniometer) - Pain intensity and interference of pain (BPI) - Pain Catastrophizing (PCS) - Functional status (HAQDI) - Perceived fatigue (FSS)

Abbreviations: EG – experimental group; CG – comparison group; MSK – musculoskeletal; IQR – interquartile range; SLR – Straight Leg Raise; ROM - Range of Motion; ULNT - Upper Limb Nerve Test; BPI – Brief Pain Inventory; PCS – Pain Catastrophizing Scale; HAQDI – Health Assessment Questionnaire Disability Index; FSS – Fatigue Severity Scale; RCT - Randomised Controlled Trial.

Full risk-of-bias assessments and summary of findings tables are presented in Supplemental Files 6, 7, 8, and 9, respectively.

### Low Back Pain

Fourteen randomised controlled trials^59,60,62–66,69–71,73,74,81,82^ and three quasi-randomised trials^58,67,68^ were included in the review (n = 701). Four trials used neural mobilisation as a single intervention^62,65,70,74^ while 13 used neural mobilisation as an element of a multimodal intervention.^58–60,63,64,66–69,71,73,81,82^ Three studies applied sliding techniques,^59,71,74^ 10 applied tensioning techniques,^58,60,63–68,73,81^ one used combined sliding and tensioning techniques,^82^ and one compared a sliding technique against a tensioning technique.^69^ Active neural mobilisation was performed in two studies,^59,82^ passive neural mobilisation was applied in one study,^81^ and combined passive and active techniques were used in 12 studies.^58,60,63–69,71,73,74^ In two studies, no details on the neural mobilisation were given.^62,70^

**Figure 2.**
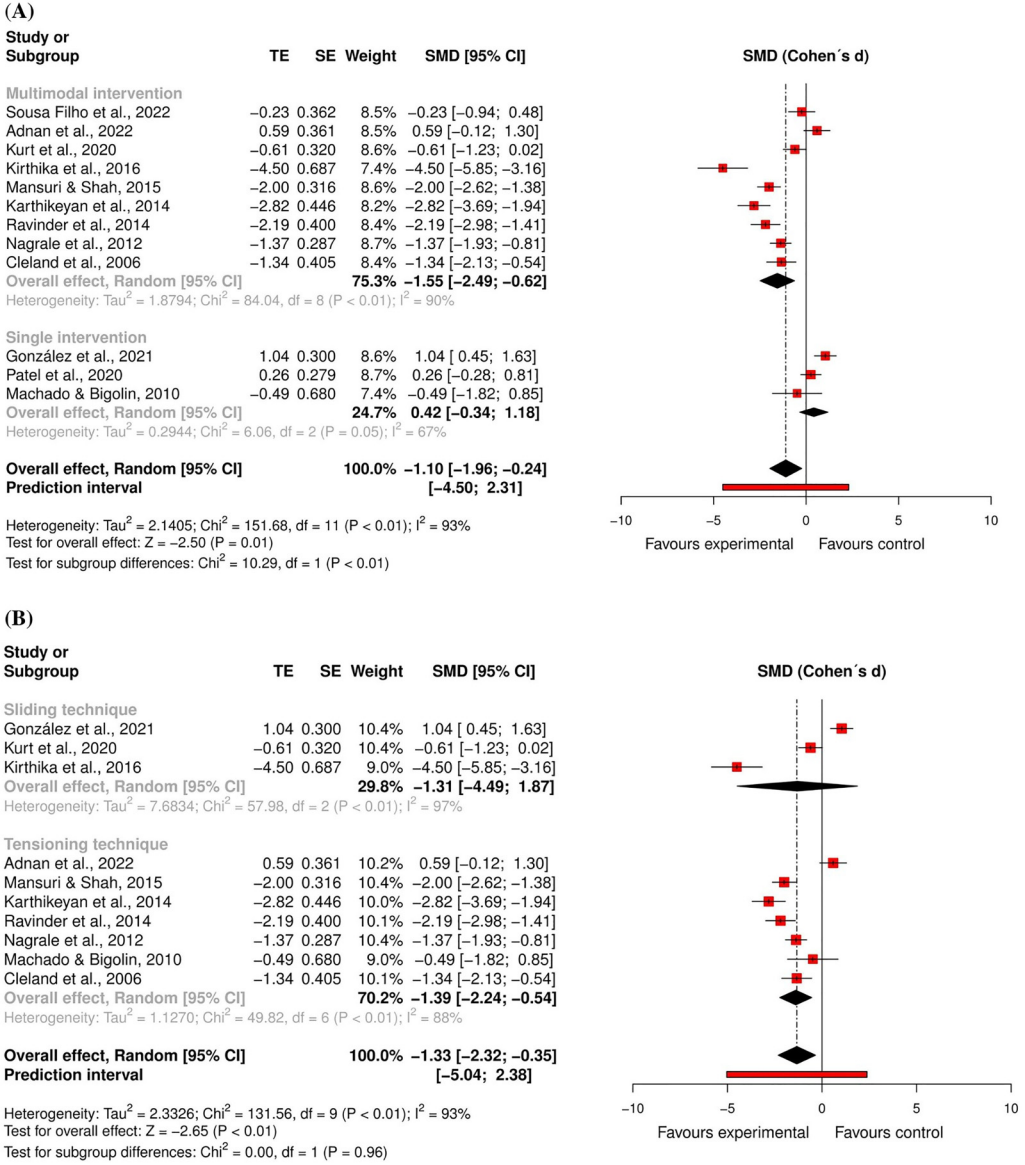
Global and subgroup standardised mean differences (95% confidence interval) in the effect of neural mobilisation versus other treatments on pain intensity in participants with low back pain. **A**: multimodal vs. single intervention; **B**: sliding vs. tensioning technique.

**Figure 3.**
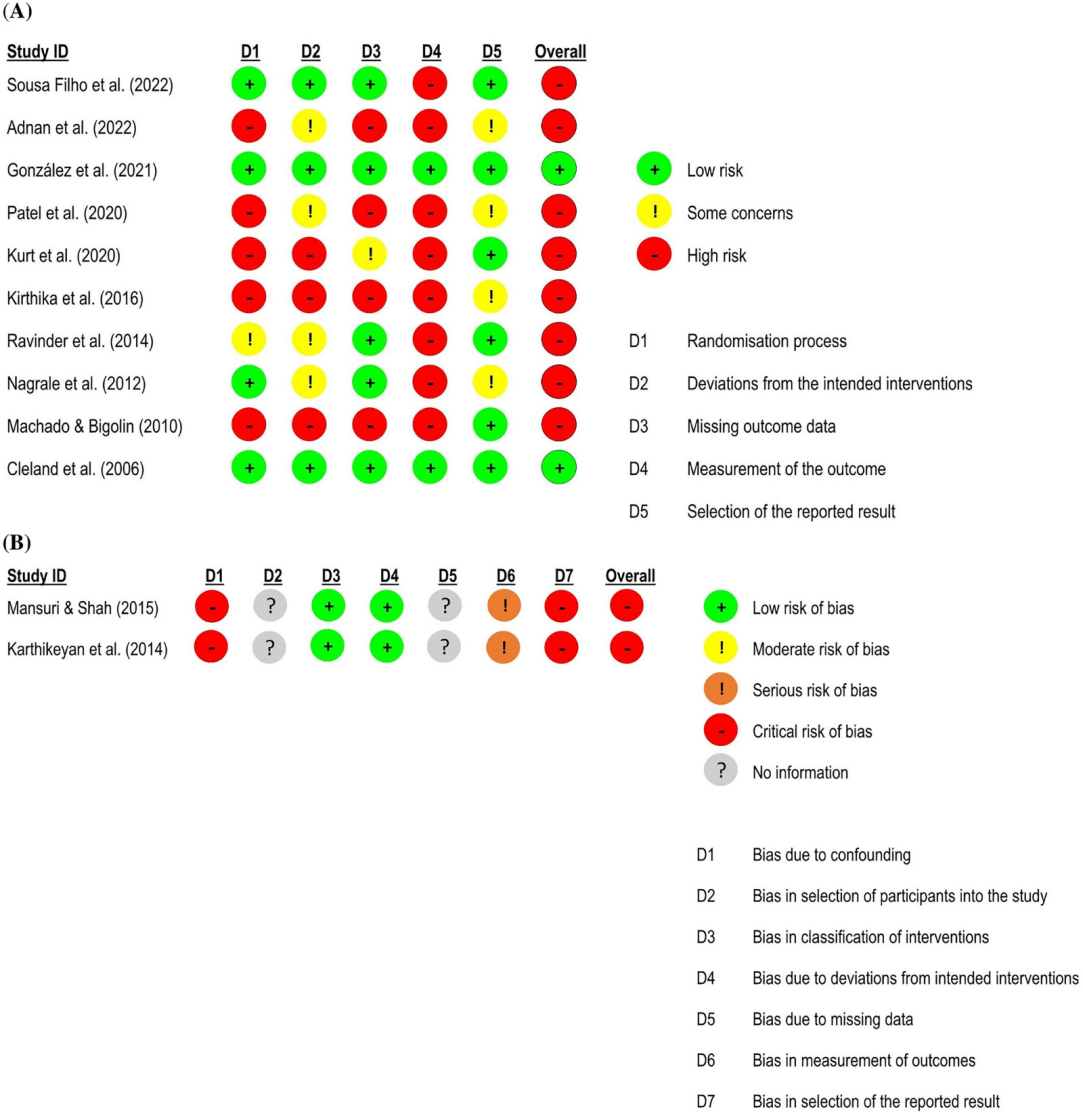
Risk of bias assessment for pain intensity in low back pain studies. **A**: RoB 2 – randomised controlled trials; **B**: ROBINS-I – quasi-randomised trials.

For *pain intensity*, 10 randomised controlled trials^59,60,62,63,66,70,71,74,81,82^ – eight at high risk of bias^59,62,63,66,70,71,81,82^ and two at low risk of bias^60,74^ – and two quasi-randomised trials at critical risk of bias^67,68^ were included in the meta-analysis (n = 476). The results showed a large and significant effect favouring the neural mobilisation group (effect size = −1.10, 95% confidence interval: −1.96; −0.24; very low certainty of evidence). A sensitivity analysis was performed excluding the study of González et al.,^74^ considering that our results differed from those reported by the original authors, probably due to the conversion of data into mean and standard deviation. However, the results remained significant and large favouring the neural mobilisation group (Supplemental File 10). Another sensitivity analysis was performed excluding studies that did not report the neural mobilisation procedures used, but the results remained significant (Supplemental File 10). Two subgroup analyses showed that neural mobilisation seems to have a large and significant effect only when integrated into a multimodal intervention (effect size = −1.55, 95% confidence interval: −2.49; −0.62) and that the overall effect is also large and significant only for tensioning techniques (effect size = −1.39, 95% confidence interval: −2.24; −0.54) (Figures 2 and 3).

**Figure 4.**
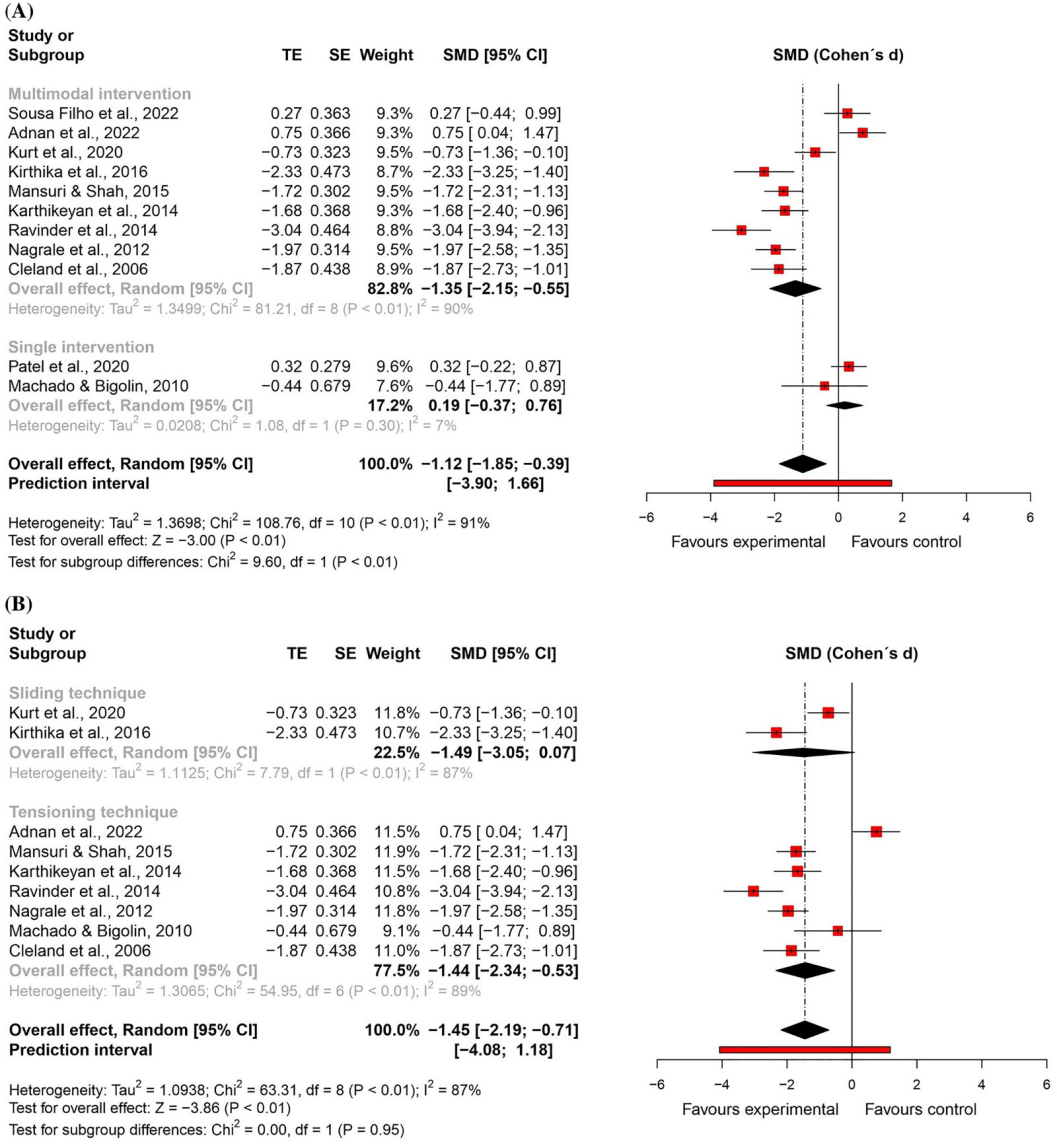
Global and subgroup standardised mean differences (95% confidence interval) in the effect of neural mobilisation versus other treatments on functional status in participants with low back pain. **A**: multimodal vs. single intervention; **B**: sliding vs. tensioning technique.

**Figure 5.**
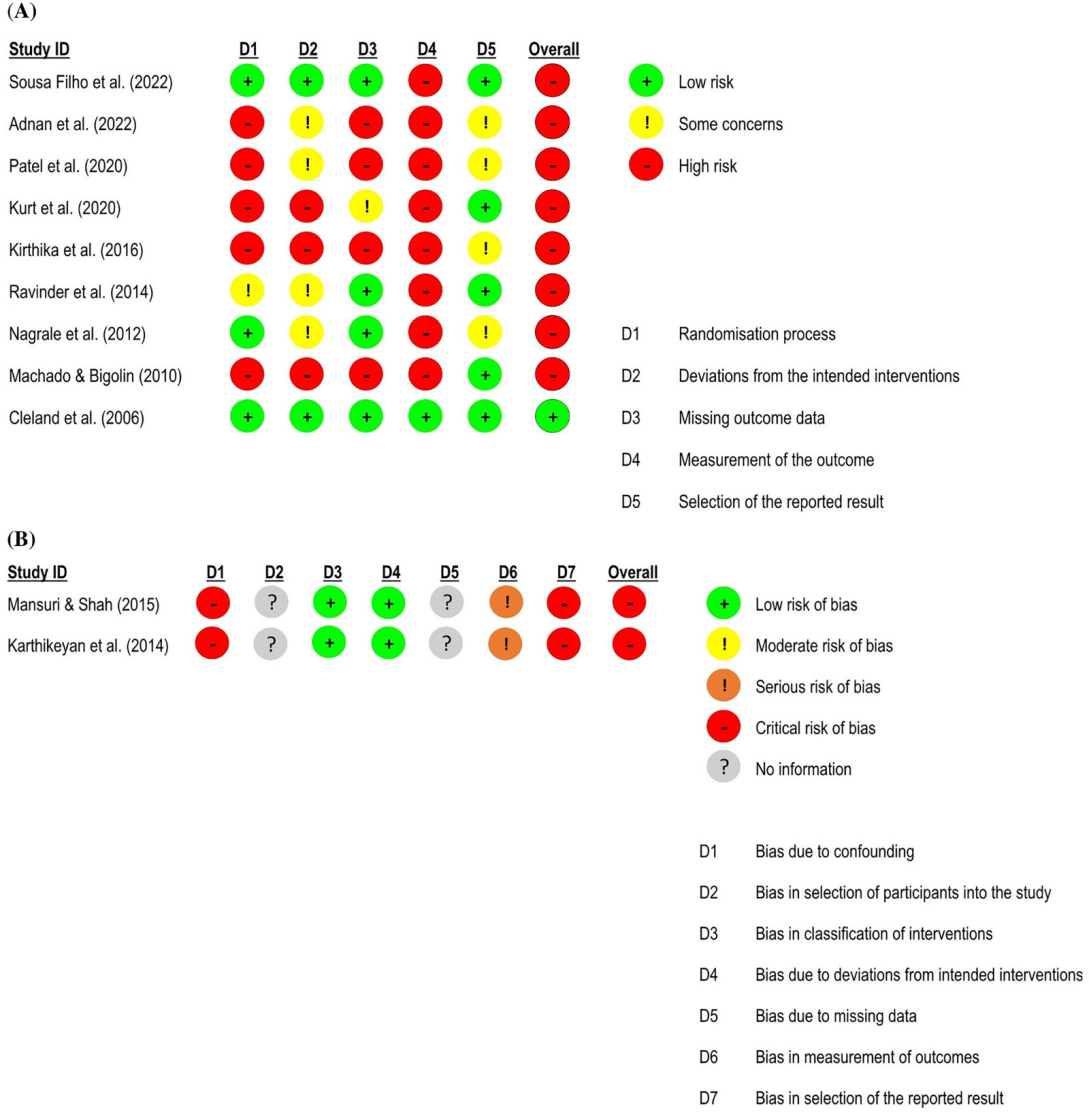
Risk of bias assessment for functional status in low back pain studies. **A**: RoB 2 – randomised controlled trials; **B**: ROBINS-I – quasi-randomised trials.

For *functional status*, nine randomised controlled trials^59,60,62,63,66,70,71,81,82^ – eight studies at high risk of bias^59,62,63,66,70,71,81,82^ and one study at low risk of bias^60^ – and two quasi-randomised trials at critical risk of bias^67,68^ were included in the meta-analysis (n = 425). The results showed a large and significant effect favouring the neural mobilisation group (effect size = −1.12, 95% confidence interval: −1.85; −0.39; very low certainty of evidence). The subgroup analyses suggest that neural mobilisation techniques appear to have a large and significant effect only when applied as part of a multimodal intervention (effect size = −1.35, 95% confidence interval: −2.15; −0.55) and with tensioning techniques (effect size = −1.44, 95% confidence interval: −2.34; −0.53) (Figures 4 and 5).

The studies carried out by Patel et al.^70^ and Sousa Filho et al.^82^ were excluded from subgroup analyses that investigated the effect of different neural mobilisation techniques (sliding *vs.* tensioning), considering that in the first, it was not possible to identify whether a sliding or tensioning technique was used and, in the second, the authors combined sliding and tensioning together.

**Figure 6.**
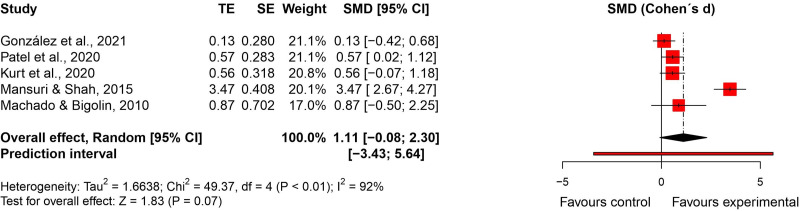
Standardised mean difference (95% confidence interval) in the effect of neural mobilisation versus other treatments on flexibility in participants with low back pain.

**Figure 7.**
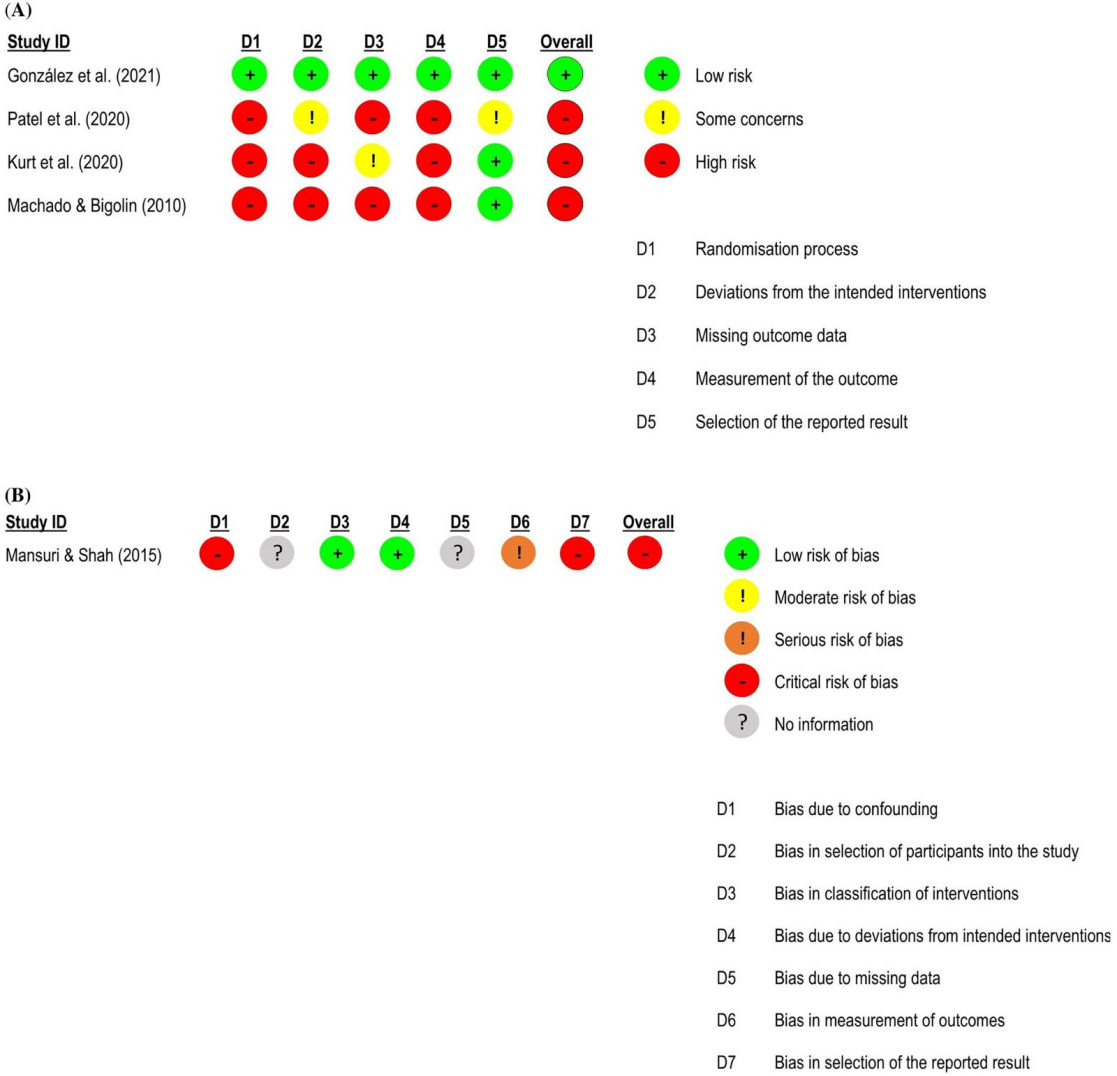
Risk of bias assessment for flexibility in low back pain studies. **A**: RoB 2 – randomised controlled trials; **B**: ROBINS-I – quasi-randomised trials.

For *flexibility*, five randomised controlled trials^59,62,70,74,81^ – four studies at high risk of bias^59,62,70,81^ and one study at low risk of bias^74^ – and one quasi-randomised trial at critical risk of bias^68^ were included in the meta-analysis (n = 245). There was no significant difference between groups (effect size = 0.87, 95% confidence interval: −0.21; 1.94; very low certainty of evidence) (Figures 6 and 7).

Five studies were not included in the meta-analysis because of insufficient data.^58,64,65,69,73^ Two studies compared different types of neural mobilisation.^64,69^ Moksha et al.,^69^ at high risk of bias for all outcomes, compared sliding neural mobilisation vs. tensioning neural mobilisation in a five-session intervention and reported a significant and large effect favouring the sliding group for pain intensity (effect size = −1.14, 95% confidence interval: −1.67; −0.60), functional status (effect size = −1.01, 95% confidence interval: −1.55; −0.47) and flexibility (effect size = 1.39, 95% confidence interval: 0.83;1.96). Malik et al.,^64^ a high risk of bias study for all outcomes, compared straight leg raise vs. slump stretching in multimodal interventions (two experimental groups) against lumbar stabilisation exercises and advice on pain intensity and flexibility in a 3-week intervention program. A significant difference favouring the neural mobilisation groups was found for flexibility, but not for pain intensity. A significant difference favouring slump stretching against straight leg raise was found for flexibility, but no difference was found between the two neural mobilisation groups for pain intensity (p > 0.05; insufficient data to calculate effect size).

Three studies (at critical and high risk of bias) compared neural mobilisation as part of a multimodal intervention against hot packs and isometric exercise,^58^ hot packs and electrotherapy,^59^ and manual therapy and exercises.^73^ No between-group significant differences were found for pain intensity, functional status, and flexibility^58^ neither for balance and gait parameters^59^ or functional status,^73^ but there was a significant between-group effect favouring the neural mobilisation group for pain intensity (p = 0.000).^73^

Three studies also evaluated outcomes at one to three weeks of follow-up.^63,73,74^ González et al.^74^ found no significant difference for pain intensity and flexibility between neural mobilisation and sham neural mobilisation. However, Jain et al.^73^ and Nagrale et al.^63^ found a significant difference favouring neural mobilisation for pain intensity and functional status when compared to spine mobilisation and exercise.

### Neck Pain

Seven randomised controlled trials^51–57^ and one crossover trial^88^ were included (n = 266). Four trials used neural mobilisation as a single intervention^52–54,56^ and other four included neural mobilisation into a multimodal intervention.^51,55,57,88^ Four studies applied sliding techniques,^52–54,57^ three applied tensioning techniques,^51,55,56^ and in one study it was not possible to identify which technique was used.^88^ Active neural mobilisation was performed in one study,^57^ passive neural mobilisation was applied in four studies,^51,55,56,88^ a combined technique (active and passive) was used in one trial,^53^ and two studies did not report on the technique used.^52,54^

**Figure 8.**
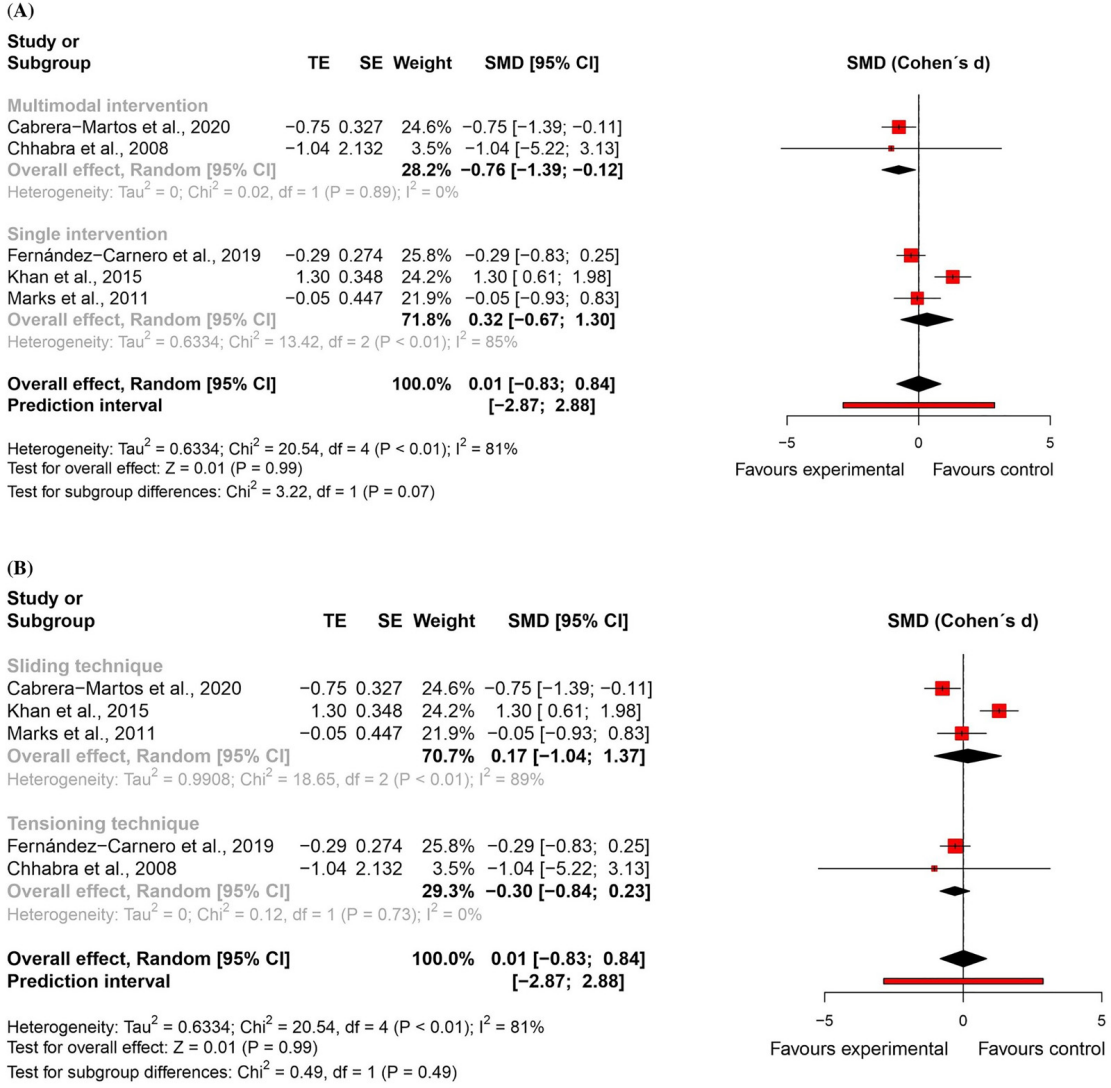
Global and subgroup standardised mean differences (95% confidence interval) in the effect of neural mobilisation versus other treatments on pain intensity in participants with neck pain. **A**: multimodal vs. single intervention; **B**: sliding vs. tensioning technique.

**Figure 9.**
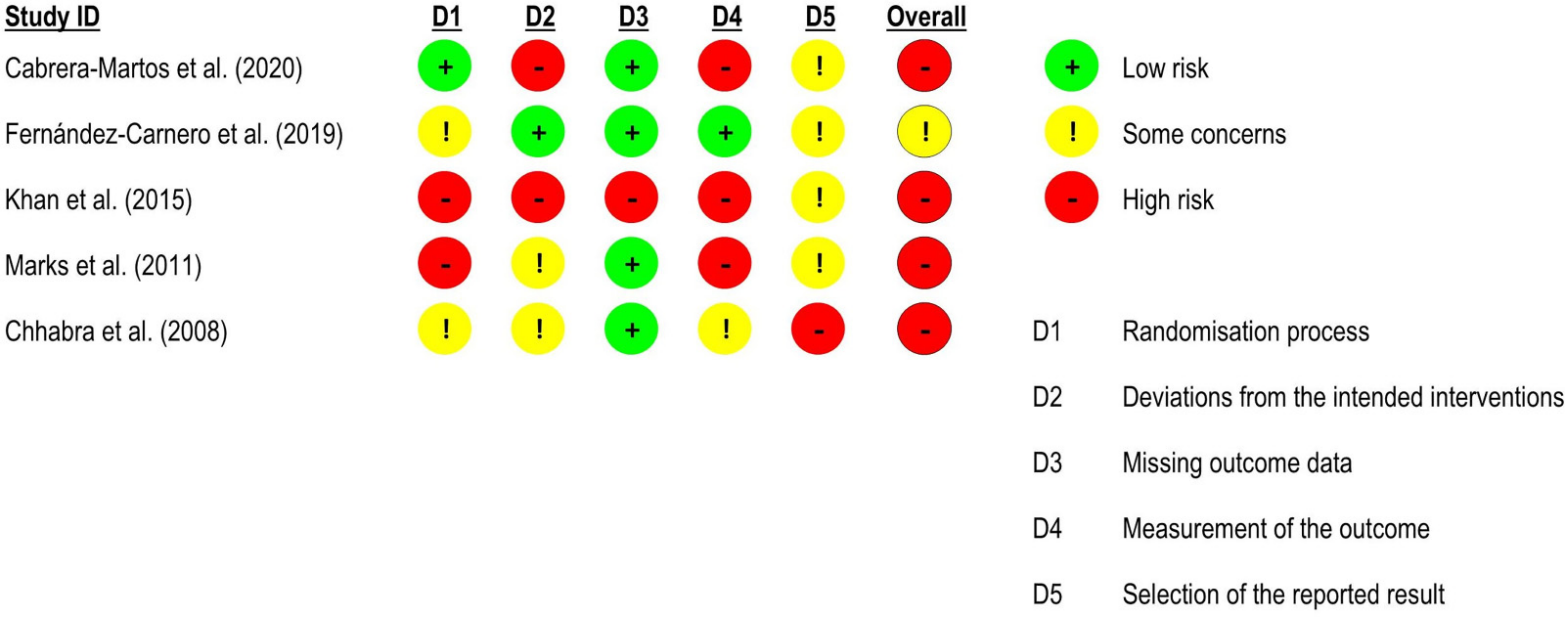
Risk of bias assessment for pain intensity in neck pain studies (RoB 2 – randomised controlled trials).

For *pain intensity*, five randomised controlled trials^51,52,54,56,57^ – four at high risk of bias^51,52,54,57^ and one with some concerns^56^ – were included in the meta-analysis (n = 191). There was no significant difference between groups (effect size = 0.01, 95% confidence interval: −0.83; 0.84; very low certainty of evidence). Subgroup analyses showed that neural mobilisation seems to have a medium and significant effect only when applied as part of a multimodal intervention (effect size = −0.76, 95% confidence interval: −1.39; −0.12) and that there was no significant difference between sliding and tensioning subgroups for pain intensity (Figures 8 and 9).

**Figure 10.**
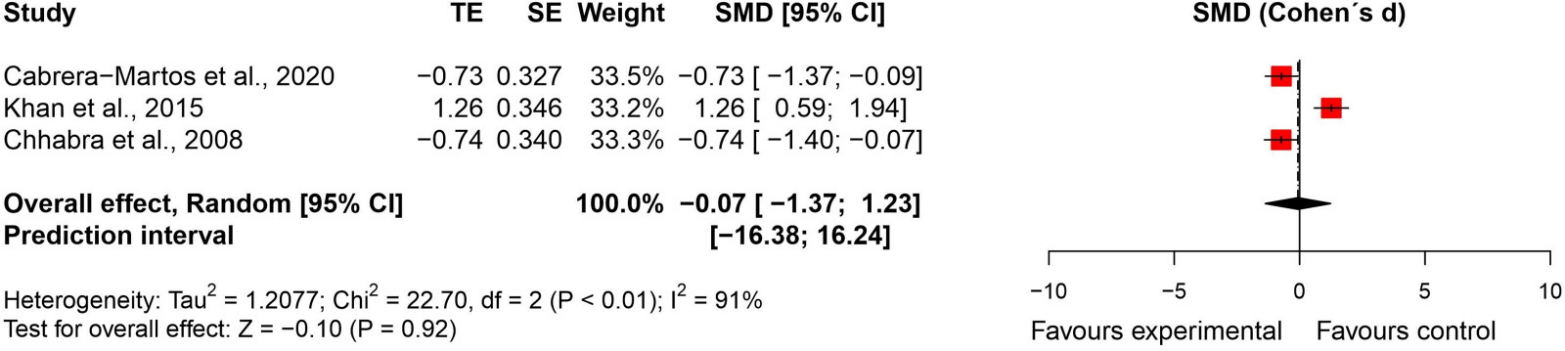
Standardised mean difference (95% confidence interval) in the effect of neural mobilisation versus other treatments on functional status in participants with neck pain.

**Figure 11.**
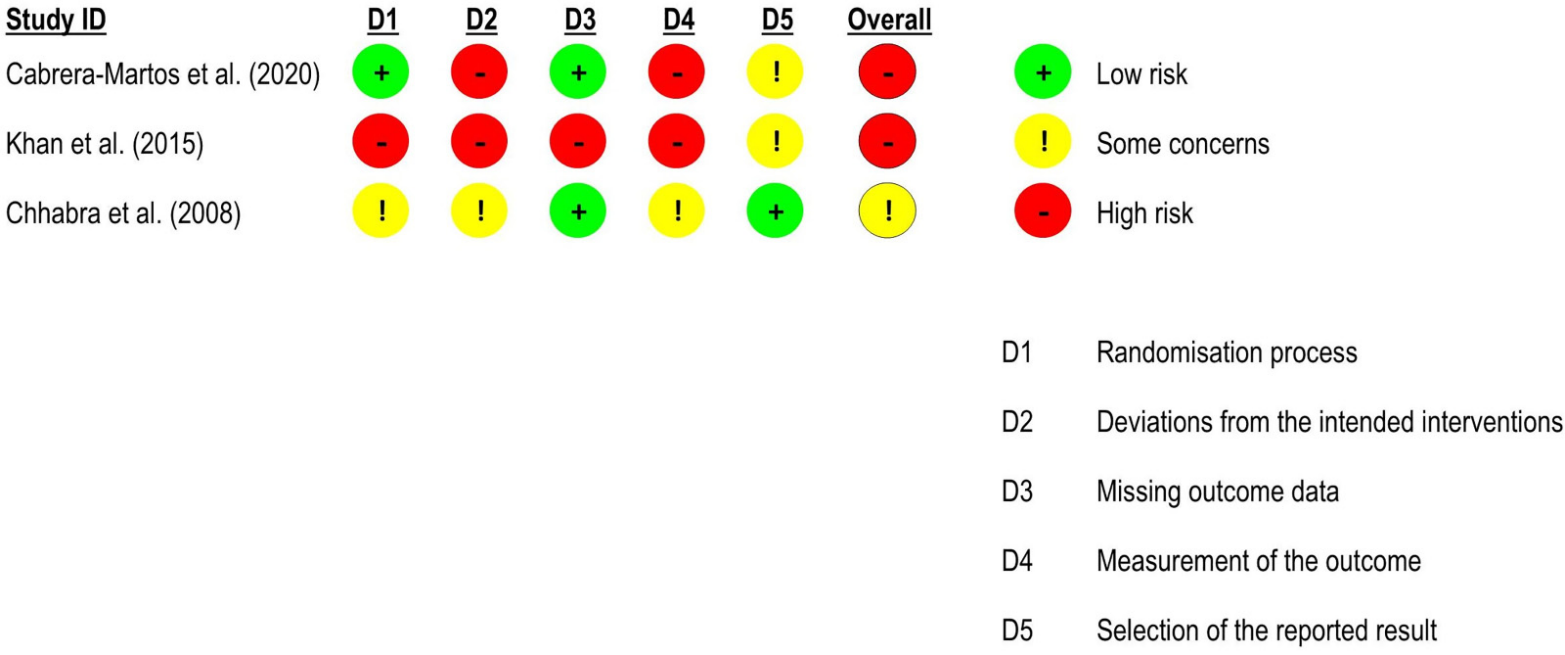
Risk of bias assessment for functional status in neck pain studies (RoB 2 – randomised controlled trials).

For *functional status*, three randomised controlled trials^51,54,57^ – two at high risk of bias^54,57^ and one with some concerns^51^ – were included in the meta-analysis (n = 117). There was no significant difference between groups (effect size = −0.07, 95% confidence interval: −1.37; 1.23; very low certainty of evidence) (Figures 10 and 11).

**Figure 12.**
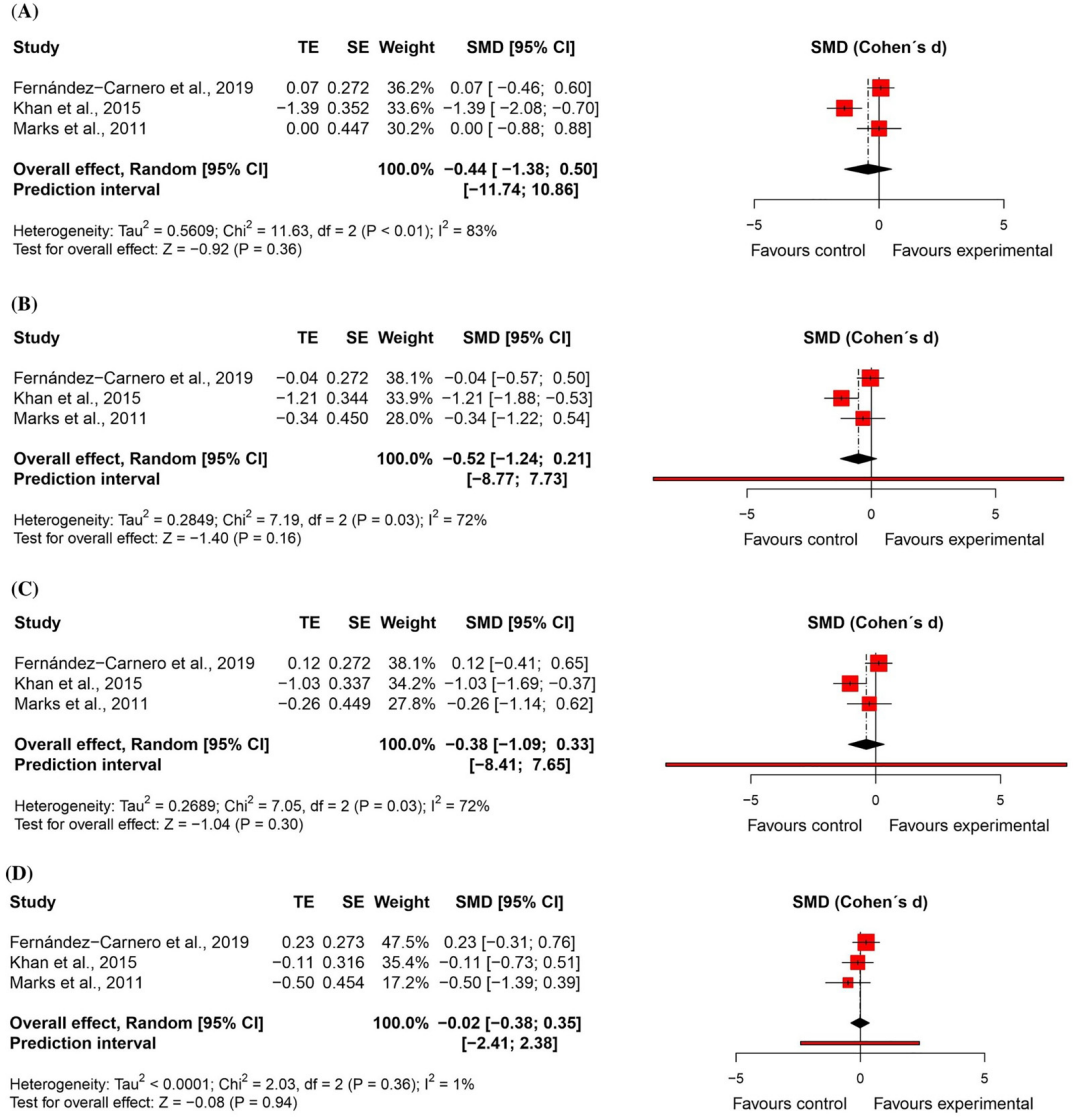
Standardised mean differences (95% confidence interval) in the effect of neural mobilisation versus other treatments on range of motion in participants with neck pain. **A**: flexion; **B**: extension; **C**: lateral flexion; **D**: rotation.

**Figure 13.**
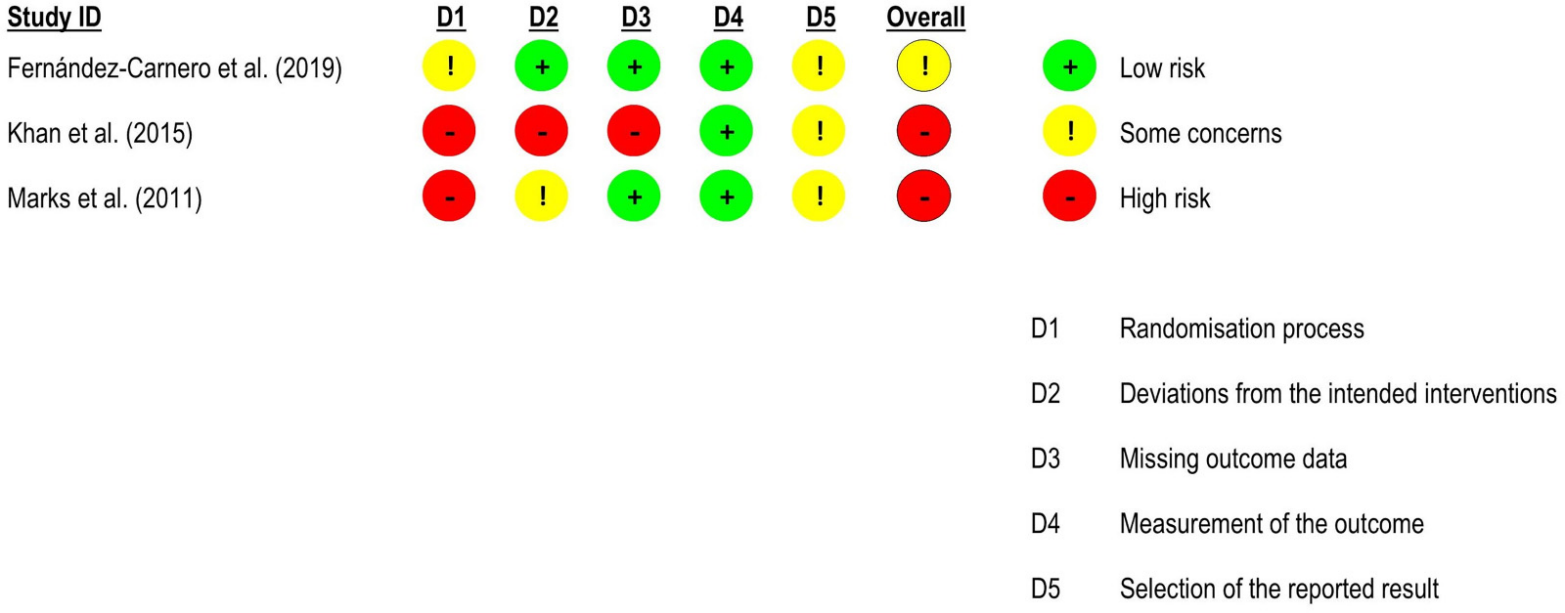
Risk of bias assessment for range of motion in neck pain studies (RoB 2 – randomised controlled trials).

For *active cervical range of motion*, three randomised controlled trials^52,54,56^ – two at high risk of bias^52,54^ and one at low risk of bias^56^ – were included in the meta-analyses (n = 114). There were no significant differences between groups (*flexion*: effect size = −0.44, 95% confidence interval: −1.38; 0.50; *extension*: effect size = −0.52, 95% confidence interval: −1.24; 0.21; *lateral flexion*: effect size = −0.38, 95% confidence interval: −1.09; 0.33; and *rotation*: effect size = −0.02, 95% confidence interval: −0.38; 0.35). All results with very low certainty of evidence (Figures 12 and 13).

Three studies at high risk of bias^53,55,88^ were not included in the meta-analysis because of insufficient data provided. Two studies compared neural mobilisation as part of a multimodal intervention against osteopathic manipulative techniques^55^ and glenohumeral/thoracic mobilisation or no intervention,^88^ respectively. A significant between-group effect favouring the neural mobilisation group was reported for pain intensity (p < 0.05) in both trials, but not for functional status and pressure pain threshold. Gupta et al.^53^ compared neural mobilisation as a single intervention against exercises plus ergonomic advice and reported significant between-group differences favouring the neural mobilisation for pain intensity and functional status (p < 0.05).

One study evaluated the results at 1-week follow-up after intervention,^52^ showing a significant effect on extension and lateral flexion range of motion favouring the comparison group (cervical mobilisation).

### Other Musculoskeletal Conditions

Three randomised controlled trials on *hand osteoarthritis*^49,50,80,87^ were included in the review (n = 192). Two trials included neural mobilisation in a multimodal intervention,^80,87^ the other used neural mobilisation as a single intervention^49,50^ and all applied passive sliding techniques. No significant difference between groups was found for pain intensity (k = 2, high risk of bias^87^ and low risk of bias^80^; effect size = −5.89, 95% confidence interval: −17.31; 5.53), pressure pain threshold in the treated hand (k = 2, some concerns^80,87^; effect size = 1.13, 95% confidence interval: −0.55; 2.81) or grip strength in the dominant limb (k = 2, low risk of bias^80^ and some concerns^87^; effect size = 0.42, 95% confidence interval: −0.28; 1.12). For pressure pain threshold in the nontreated hand, significant differences between groups were found at post-intervention (k = 3, with some concerns for risk of bias^49,80,87^; effect size = 0.83, 95% confidence interval: 0.07; 1.59) and at two months follow-up (k = 2; effect size = 0.61, 95% confidence interval: 0.05; 1.17).

Two randomised controlled trials,^61,84^ one quasi-randomised trial,^86^ and one crossover trial^83^ on *lateral epicondylitis* were included (n = 108). Three trials included neural mobilisation in a multimodal intervention,^61,84,86^ while one study used neural mobilisation as a single intervention.^83^ Three studies applied tensioning techniques^61,83,84^ and one study used sliding and tensioning techniques combined.^86^ No significant difference between groups was found for pain intensity (k = 2, high risk of bias^61^ and critical risk of bias^86^; effect size = −0.42, 95% confidence interval: −1.18; 0.34), functional status (k = 3, high risk of bias^61,84^ and critical risk of bias^86^; effect size = −0.18, 95% confidence interval: −0.94; 0.58) and grip strength (k = 3, high risk of bias^61,84^ and critical risk of bias^86^; effect size = 0.53, 95% confidence interval: −0.04; 1.11). Two studies^61,84^ also assessed grip strength at two to three months follow-up and no significant difference between groups was found (effect size = −0.16, 95% confidence interval: −0.51; 0.18). For pressure pain threshold*,* one quasi-randomised trial^86^ (at critical risk of bias) showed a medium and significant effect favouring neural mobilisation (effect size = 0.73, 95% confidence interval: 0.09; 1.37). Another high risk of bias study^83^ reported large and significant differences for 24 h pain scores between the neural mobilisation and the placebo groups (effect size = −5.12, 95% confidence interval: −7.02; −3.22) and between neural mobilisation and the no intervention groups (effect size = −6.28, 95% confidence interval: −8.59; −3.98) favouring the neural mobilisation group. The authors also stated that there were significant differences favouring the neural mobilisation group for grip strength and pressure pain threshold (p < 0.05), but data provided were insufficient for effect size calculation.

Two randomised controlled trials^78,79^ on *rheumatoid arthritis* compared neural mobilisation as a single intervention against joint mobilisation exercises in a 4-week intervention (n = 30). One randomised controlled trial^78^ applied tensioning techniques, while the other^79^ used tensioning and sliding techniques combined. Both studies used active techniques. No significant difference between groups was found for pain intensity (k = 2, high risk of bias^78,79^; effect size = −0.43, 95% confidence interval: −1.16; 0.29). Lau et al.^78^ also assessed the functional status and reported no significant difference between groups.

One randomised controlled trial^72^ on *ankle sprain*, with some concerns about the risk of bias for all outcomes, compared a 4-week intervention of neural mobilisation (tensioning and passive techniques) as part of a multimodal intervention against proprioceptive/strengthening exercises for pain intensity, functional ankle instability, pressure pain threshold, muscle strength, and active range of motion in participants with recurrent ankle sprains (n = 56). There were large and significant differences favouring the neural mobilisation group for pain intensity (effect size = −1.21, 95% confidence interval: −1.78; −0.64), functional ankle instability (effect size = 1.39, 95% confidence interval: 0.80; 1.97), pressure pain threshold (effect size = 0.85, 95% confidence interval: 0.31; 1.40), flexor muscles strength (effect size = 4.84, 95% confidence interval: 3.80; 5.88), extensor muscles strength (effect size = 3.30, 95% confidence interval: 2.49; 4.10), flexion range of motion (effect size = 1.76, 95% confidence interval: 1.14; 2.38), and extension range of motion (effect size = 2.25, 95% confidence interval: 1.58; 2.92).

One randomised controlled trial^85^ on *shoulder impingement syndrome*, at high risk of bias for both outcomes measured, compared a 5-week intervention of neural mobilisation (sliding and tensioning techniques combined and active and passive techniques combined) as part of a multimodal intervention against electrotherapy and exercises for pain intensity and functional status (n = 80). There were large and significant differences favouring the neural mobilisation group for pain intensity (effect size = −1.89, 95% confidence interval: −2.41; −1.36) and functional status (effect size = 2.48, 95% confidence interval: 1.90; 3.07).

One randomised controlled trial^75^ on *plantar heel pain syndrome*, at high risk of bias for both outcomes measured, compared neural mobilisation (tensioning and active techniques) in a multimodal intervention against ultrasound and exercises for pain intensity and functional status in a 4 to 6-week intervention (n = 69). There was only a medium and significant difference favouring the neural mobilisation group for functional status (effect size = 0.55, 95% confidence interval: 0.07; 1.04).

One randomised controlled trial^77^ on *unspecified musculoskeletal pain*, at low risk of bias for all outcomes, investigated the effectiveness of neural mobilisation (sliding active techniques) in a multimodal intervention against exercises on pain intensity, flexibility, balance, gait, and mobility in an 8-week intervention program in older adults (n = 26). They found no between group significant differences for any of the variables.

One randomised controlled trial^76^ on *fibromyalgia*, at high risk of bias for all outcomes, compared neural mobilisation (sliding, tensioning, and active techniques combined) as a single intervention against usual activities and advice on pain intensity, functional status, and range of motion of neurodynamic tests after an 8-week intervention (n = 48). There were medium effect size for functional status (effect size = 0.67, 95% confidence interval: 0.09; 1.25) and Upper Limb Nerve Test 1 range of motion (effect size = 0.63, 95% confidence interval: 0.05; 1.21) and large effect size for pain intensity (effect size = −0.93, 95% confidence interval: −1.53; −0.34), slump stretching range of motion (effect size = 0.87, 95% confidence interval: 0.28; 1.46), Upper Limb Nerve Test 2a range of motion (effect size = 0.86, 95% confidence interval: 0.26; 1.45]), and Upper Limb Nerve Test 2b range of motion (effect size = 1.00, 95% confidence interval: 0.40; 1.60) favouring the neural mobilisation group.

None of the included studies reported data related to other secondary outcomes, such as morphological and functional changes in peripheral nerves and neurophysiological changes.

Effect sizes for pain intensity and functional status from studies of other musculoskeletal conditions are available in two forest plots in Supplemental File 11, where overall effects have been removed.

## Discussion

This systematic review showed that neural mobilisation may have positive effects on pain and function for patients with low back pain and on pain for patients with neck pain when integrated into multimodal interventions. However, there were no significant differences favouring neural mobilisation in improving the flexibility and range of motion for both conditions. All results have very low certainty of evidence.

For low back pain, the meta-analyses suggest that neural mobilisation contributes to pain decrease and functional status improvement, which corroborates findings of previous reviews.^8,15,89^ However, these results seem to depend on whether neural mobilisation is administered as part of a multimodal intervention and the type of neural mobilisation being applied. A significant effect favouring tensioning techniques but not sliding techniques conflicts with the findings of a recent study that compared the neurophysiological responses of sliding and tensioning and reported the tensioning technique to have adverse effects on the nerves, such as decreased amplitude of the dermatomal somatosensory evoked potential.^90^ In addition, one of the studies included in this review, but not in the meta-analysis, compared the sliding against tensioning neural mobilisation and reported significant results favouring the sliding technique for both pain intensity and functional status.^69^ Another trial, not included in this review because it included participants with neuropathic pain, found no differences between the two techniques for pain intensity.^91^ According to Coppieters et al.,^10^ sliding techniques are less aggressive and may be indicated for acute conditions, as they reduce the possibility of causing nerve irritation and inflammation. On the other hand, tensioning techniques, despite being more ‘aggressive,’ can have beneficial effects through a nerve pumping action, reducing intraneural pressure and improving circulation.^10^ Therefore, considering that only three studies^59,71,74^ included in the meta-analysis used sliding neural mobilisation and that some trials included people with chronic pain while others included people with acute/subacute pain, caution is needed when interpreting this subgroup analysis. Regarding flexibility, there was no between group significant difference, unlike what was shown by Neto et al.^89^ who investigated the effectiveness of neural mobilisation on flexibility in a healthy population,^89^ suggesting that musculoskeletal pain can play an important role in flexibility.^92^

For neck pain, similarly to low back pain, the results suggest a difference between groups favouring neural mobilisation in decreasing pain when applied as part of a multimodal intervention, but not as a single intervention, which is in line with a previous review.^19^ Other interventions have also been found to be effective for pain intensity only when included into a multimodal physiotherapy intervention.^93–96^ For functional status and cervical range of motion, there were no significant differences between groups, which agrees with the findings of Varangot-Reille et al.^19^

It was not possible to perform meta-analyses for the other musculoskeletal conditions and outcomes, such as pressure pain threshold and muscle strength, which limits the conclusions to be drawn. González-Matilla et al.^18^ identified an effectiveness of neural mobilisation when compared to joint mobilisation and exercises for pain intensity in people with autoinflammatory diseases (hand osteoarthritis and rheumatoid arthritis), but they showed a pooled global effect for both conditions.^87^ Our analyses revealed no statistical significance for pain improvement in any of these conditions individually. Gamelas et al.^13^ found a significant effect of neural mobilisation tensioning technique on pressure pain threshold, but in asymptomatic individuals. Regarding muscle strength, the results are conflicting in the literature.^13,97–99^ In this systematic review, we could only assess grip strength in a few studies.

Several limitations were identified in our review. Most studies have a high or critical risk of bias. Few studies were available for some musculoskeletal conditions with small sample sizes, potentially impacting the accuracy of the estimates. In addition, high methodological heterogeneity between trials (e.g., different types of comparison groups, patient characteristics) hampers the generalisability, transferability, and applicability of the results. Also, most studies did not assess the underlying predominant mechanism of pain hindering a mechanism-based analysis. Furthermore, many studies did not describe the intervention in sufficient detail (e.g., dose and frequency of intervention, the neural mobilisation technique used [sliding, tensioning, passive, active]), which makes it difficult to establish a relationship between the effectiveness of neural mobilisation and specific doses and characteristics of the intervention. Given all these limitations, the evidence remains unclear for all outcomes evaluated.

Regarding the clinical management of low back and neck pain, the results found suggest that neural mobilisation can be effective when administered as part of a multimodal intervention, where most studies applied the technique for 6 to 12 sessions. In addition, tensioning techniques seem to be more effective in decreasing pain and disability compared to sliding techniques for individuals with low back pain. The frequency of treatment across studies ranged from protocols performed twice a week under supervision to protocols performed daily at home. The duration of application of neural mobilisation within a multimodal intervention protocol ranged from 5 to 20 min, although many authors did not report the duration of the treatment protocol. Most studies on low back pain used slump-stretching exercises with a dose of three to five repetitions of 30 s each, while studies on neck pain used nerve mobilisation exercises for the upper limbs with a dose of three sets of 10 repetitions for each exercise. However, it should be considered that, according to the GRADE assessment performed, these recommendations have a very low certainty of evidence, therefore, new clinical trials should explore the effectiveness of different doses, types, and duration of neural mobilisation programs to better inform clinical practice. Taking into account the other musculoskeletal conditions, it was not possible to establish specific recommended doses, considering the small number of trials included.

To the best of our knowledge, no previous systematic review has explored the comparison of different types of neural mobilisation (sliding, tensioning, passive, and active) on pain intensity and/or functional status in people with musculoskeletal pain, so future clinical trials may enrich these analyses in larger samples. New studies should also assess participantś predominant pain mechanism, considering the updated paradigm on pain assessment and management for different pain phenotypes.^100–103^ In addition, future studies should describe the intervention in detail, as well as to perform follow-up evaluations to verify the duration of effect, as few studies have performed these assessments.

In summary, neural mobilisation techniques seem to have a positive impact on improving pain and functional status in people with low back pain and on improving pain in people with neck pain when applied as part of a multimodal intervention program. Tensioning techniques appear to be more effective in improving pain and function in people with low back pain when compared to sliding techniques. No significant effects were found for flexibility and range of motion for both conditions. Regarding other musculoskeletal conditions, very few studies were included in the review, therefore, it is not possible to conclude whether neural mobilisation is effective in improving pain and function, as well as secondary variables (pressure pain threshold and grip muscle strength). None of the studies reported data on immune responses and neural morphological and neurophysiological changes. There was very low confidence for all effect estimates. New studies with more robust methodological procedures are needed to confirm the findings.

**Clinical messages**

- Neural mobilisation helps decrease low back and neck pain when integrated into multimodal interventions.
- Tensioning techniques appear to be effective in treating low back pain, but sliding techniques are not.
- Slump-stretching exercises and upper limbs nerve mobilisation can be included in low back and neck pain management programs, respectively.

## Supplemental Material

sj-docx-1-cre-10.1177_02692155231215216 - Supplemental material for Effectiveness of Neural Mobilisation on Pain Intensity, Functional Status, and Physical Performance in Adults with Musculoskeletal Pain – A Systematic Review with Meta-Analysis Click here for additional data file. Supplemental material, sj-docx-1-cre-10.1177_02692155231215216 for Effectiveness of Neural Mobilisation on Pain Intensity, Functional Status, and Physical Performance in Adults with Musculoskeletal Pain – A Systematic Review with Meta-Analysis by Frederico Mesquita Baptista, Ellen Nery, Eduardo Brazete Cruz, Vera Afreixo and Anabela G Silva in Clinical Rehabilitation

sj-xlsx-2-cre-10.1177_02692155231215216 - Supplemental material for Effectiveness of Neural Mobilisation on Pain Intensity, Functional Status, and Physical Performance in Adults with Musculoskeletal Pain – A Systematic Review with Meta-Analysis Click here for additional data file. Supplemental material, sj-xlsx-2-cre-10.1177_02692155231215216 for Effectiveness of Neural Mobilisation on Pain Intensity, Functional Status, and Physical Performance in Adults with Musculoskeletal Pain – A Systematic Review with Meta-Analysis by Frederico Mesquita Baptista, Ellen Nery, Eduardo Brazete Cruz, Vera Afreixo and Anabela G Silva in Clinical Rehabilitation

sj-docx-3-cre-10.1177_02692155231215216 - Supplemental material for Effectiveness of Neural Mobilisation on Pain Intensity, Functional Status, and Physical Performance in Adults with Musculoskeletal Pain – A Systematic Review with Meta-Analysis Click here for additional data file. Supplemental material, sj-docx-3-cre-10.1177_02692155231215216 for Effectiveness of Neural Mobilisation on Pain Intensity, Functional Status, and Physical Performance in Adults with Musculoskeletal Pain – A Systematic Review with Meta-Analysis by Frederico Mesquita Baptista, Ellen Nery, Eduardo Brazete Cruz, Vera Afreixo and Anabela G Silva in Clinical Rehabilitation

sj-xlsx-4-cre-10.1177_02692155231215216 - Supplemental material for Effectiveness of Neural Mobilisation on Pain Intensity, Functional Status, and Physical Performance in Adults with Musculoskeletal Pain – A Systematic Review with Meta-Analysis Click here for additional data file. Supplemental material, sj-xlsx-4-cre-10.1177_02692155231215216 for Effectiveness of Neural Mobilisation on Pain Intensity, Functional Status, and Physical Performance in Adults with Musculoskeletal Pain – A Systematic Review with Meta-Analysis by Frederico Mesquita Baptista, Ellen Nery, Eduardo Brazete Cruz, Vera Afreixo and Anabela G Silva in Clinical Rehabilitation

sj-xlsx-5-cre-10.1177_02692155231215216 - Supplemental material for Effectiveness of Neural Mobilisation on Pain Intensity, Functional Status, and Physical Performance in Adults with Musculoskeletal Pain – A Systematic Review with Meta-Analysis Click here for additional data file. Supplemental material, sj-xlsx-5-cre-10.1177_02692155231215216 for Effectiveness of Neural Mobilisation on Pain Intensity, Functional Status, and Physical Performance in Adults with Musculoskeletal Pain – A Systematic Review with Meta-Analysis by Frederico Mesquita Baptista, Ellen Nery, Eduardo Brazete Cruz, Vera Afreixo and Anabela G Silva in Clinical Rehabilitation

sj-xlsm-6-cre-10.1177_02692155231215216 - Supplemental material for Effectiveness of Neural Mobilisation on Pain Intensity, Functional Status, and Physical Performance in Adults with Musculoskeletal Pain – A Systematic Review with Meta-Analysis Click here for additional data file. Supplemental material, sj-xlsm-6-cre-10.1177_02692155231215216 for Effectiveness of Neural Mobilisation on Pain Intensity, Functional Status, and Physical Performance in Adults with Musculoskeletal Pain – A Systematic Review with Meta-Analysis by Frederico Mesquita Baptista, Ellen Nery, Eduardo Brazete Cruz, Vera Afreixo and Anabela G Silva in Clinical Rehabilitation

sj-xlsx-7-cre-10.1177_02692155231215216 - Supplemental material for Effectiveness of Neural Mobilisation on Pain Intensity, Functional Status, and Physical Performance in Adults with Musculoskeletal Pain – A Systematic Review with Meta-Analysis Click here for additional data file. Supplemental material, sj-xlsx-7-cre-10.1177_02692155231215216 for Effectiveness of Neural Mobilisation on Pain Intensity, Functional Status, and Physical Performance in Adults with Musculoskeletal Pain – A Systematic Review with Meta-Analysis by Frederico Mesquita Baptista, Ellen Nery, Eduardo Brazete Cruz, Vera Afreixo and Anabela G Silva in Clinical Rehabilitation

sj-xlsm-8-cre-10.1177_02692155231215216 - Supplemental material for Effectiveness of Neural Mobilisation on Pain Intensity, Functional Status, and Physical Performance in Adults with Musculoskeletal Pain – A Systematic Review with Meta-Analysis Click here for additional data file. Supplemental material, sj-xlsm-8-cre-10.1177_02692155231215216 for Effectiveness of Neural Mobilisation on Pain Intensity, Functional Status, and Physical Performance in Adults with Musculoskeletal Pain – A Systematic Review with Meta-Analysis by Frederico Mesquita Baptista, Ellen Nery, Eduardo Brazete Cruz, Vera Afreixo and Anabela G Silva in Clinical Rehabilitation

sj-pdf-9-cre-10.1177_02692155231215216 - Supplemental material for Effectiveness of Neural Mobilisation on Pain Intensity, Functional Status, and Physical Performance in Adults with Musculoskeletal Pain – A Systematic Review with Meta-Analysis Click here for additional data file. Supplemental material, sj-pdf-9-cre-10.1177_02692155231215216 for Effectiveness of Neural Mobilisation on Pain Intensity, Functional Status, and Physical Performance in Adults with Musculoskeletal Pain – A Systematic Review with Meta-Analysis by Frederico Mesquita Baptista, Ellen Nery, Eduardo Brazete Cruz, Vera Afreixo and Anabela G Silva in Clinical Rehabilitation

sj-docx-10-cre-10.1177_02692155231215216 - Supplemental material for Effectiveness of Neural Mobilisation on Pain Intensity, Functional Status, and Physical Performance in Adults with Musculoskeletal Pain – A Systematic Review with Meta-Analysis Click here for additional data file. Supplemental material, sj-docx-10-cre-10.1177_02692155231215216 for Effectiveness of Neural Mobilisation on Pain Intensity, Functional Status, and Physical Performance in Adults with Musculoskeletal Pain – A Systematic Review with Meta-Analysis by Frederico Mesquita Baptista, Ellen Nery, Eduardo Brazete Cruz, Vera Afreixo and Anabela G Silva in Clinical Rehabilitation

sj-docx-11-cre-10.1177_02692155231215216 - Supplemental material for Effectiveness of Neural Mobilisation on Pain Intensity, Functional Status, and Physical Performance in Adults with Musculoskeletal Pain – A Systematic Review with Meta-Analysis Click here for additional data file. Supplemental material, sj-docx-11-cre-10.1177_02692155231215216 for Effectiveness of Neural Mobilisation on Pain Intensity, Functional Status, and Physical Performance in Adults with Musculoskeletal Pain – A Systematic Review with Meta-Analysis by Frederico Mesquita Baptista, Ellen Nery, Eduardo Brazete Cruz, Vera Afreixo and Anabela G Silva in Clinical Rehabilitation
