# Supplementary material for: Effectiveness of Neural Mobilisation on Pain Intensity, Functional Status, and Physical Performance in Adults with Musculoskeletal Pain – A Systematic Review with Meta-Analysis
Source: Clin Rehabil. 2023 Nov 21;38(2):145–83. doi: 10.1177/02692155231215216 (PMC10725147; doi:10.1177/02692155231215216)
Supplement: sj-docx-1-cre-10.1177_02692155231215216 - Supplemental material for Effectiveness of Neural Mobilisation on Pain Intensity, Functional Status, and Physical Performance in Adults with Musculoskeletal Pain – A Systematic Review with Meta-Analysis [file sj-docx-1-cre-10.1177_02692155231215216.docx]

**Supplemental File 1 – Protocol amendments**

| **Section amended** | **Protocol version** | **Systematic review version** | **Justification** |
| --- | --- | --- | --- |
| Eligibility criteria | Population – inclusion of diseases of the nervous system; no mention of people with post-surgical pain | Population – exclusion of people with neurological deficits and with post-surgical pain | The choice to exclude people with neurological impairments was based on what was found in previous exploratory research in the literature. Previous systematic reviews on the effectiveness of NM techniques have mainly prioritized the inclusion of participants with nerve-related pain). Therefore, the effectiveness of NM in the management of musculoskeletal pain where the symptom related to nervous tissue does not seem to be the main characteristic of the condition is still unclear. Regarding post-surgical pain, considering that there is relevant external interference in surgical procedures that can influence pain mechanisms, structures other than musculoskeletal ones may be involved. |
| Eligibility criteria | Study design – exclusion of crossover trials | Study design – inclusion of crossover trials | To make our search as comprehensive as possible, we decided to include crossover trials in the review, but they were excluded from the meta-analyses. |
| Search strategy | See “S2 Appendix” – PEDro database in the protocol publication^19^ | See “Supplemental File 2” – PEDro database in actual document | Considering the functionality characteristics of the PEDro database, we changed the search strategy (previously presented in the protocol version) to make the search more restricted and specific to our research question. |
| Search strategy | Covidence Systematic Review software was cited as the tool that would be used to facilitate collaboration between reviewers throughout the study selection process. | Instead, we used the CADIMA platform (CADIMA v. 2.2.3, Julius Kuehn-Institute [JKI], Federal Research Centre for Cultivated Plants, Quedlinburg, Germany, 2021). | Covidence systematic review software could not be accessed. |
| Study risk of bias assessment | The ´effect of adhering´ to intervention (the “per-protocol effect”) had been established as the effect of  interest. | The ´effect of assignment´ to intervention (ITT analysis) was considered for the systematic review. | An ITT analysis retains the benefit of randomization that, generally, the groups being compared are similar at baseline regarding measured or unmeasured prognostic factors^31^. |
