# Supplementary material for: Effectiveness of Neural Mobilisation on Pain Intensity, Functional Status, and Physical Performance in Adults with Musculoskeletal Pain – A Systematic Review with Meta-Analysis
Source: Clin Rehabil. 2023 Nov 21;38(2):145–83. doi: 10.1177/02692155231215216 (PMC10725147; doi:10.1177/02692155231215216)
Supplement: sj-docx-3-cre-10.1177_02692155231215216 - Supplemental material for Effectiveness of Neural Mobilisation on Pain Intensity, Functional Status, and Physical Performance in Adults with Musculoskeletal Pain – A Systematic Review with Meta-Analysis [file sj-docx-3-cre-10.1177_02692155231215216.docx]

**Supplemental File 3 – Individual study results**

Table 1 – Results of individual trials included in the qualitative synthesis (Low back pain)

| Study ID | Between-group difference (95% CI) | p-value (of the between-group difference) | Effect size (95% CI) |
| --- | --- | --- | --- |
| Moksha et al. (2019) | **Pain intensity (VAS)**  -1.17 (-1.6; -0.6)*  *favouring the sliding technique*  **Functional status (MODI)**  -7.45 (-11.26; -3.63)*  *favouring the sliding technique*  **Flexibility (SLR test)**  10.4 (6.55; 14.28)*  *favouring the sliding technique* | **Pain intensity**  p < 0.001*  *favouring the sliding technique*  **Functional status**  p < 0.001*  *favouring the sliding technique*  **Flexibility**  p < 0.001*  *favouring the sliding technique* | **Pain intensity**  ES = -1.14 [95%-CI -1.67; -0.60]*  *favouring the sliding technique*  **Functional status**  ES = -1.01 [95%-CI -1.55; -0.47]*  *favouring the sliding technique*  **Flexibility**  ES = 1.39 [95%-CI 0.83; 1.96]*  *favouring the sliding technique* |
| Jaidka et al. (2016) | There were no significant differences between groups for pain intensity, functional status, and flexibility (values could not be calculated). | p > 0.05 (for all outcomes) | Could not be calculated |
| Patel et al. (2014) | There were no significant differences between groups for pain intensity and flexibility (values could not be calculated). | **Pain intensity**  p = 0.1155  **Flexibility**  p = 0.0983 | Could not be calculated |
| Jain et al. (2012) | There was a significant difference between groups only for pain intensity (values could not be calculated). | **Pain intensity**  p = 0.000*  *favouring the NM group*  **Functional status**  p = 0.0865 | Could not be calculated |
| Malik et al. (2012) | There was a significant difference between groups only for flexibility (values could not be calculated). | **Pain intensity**  p > 0.05  **Flexibility**  P < 0.05*  *favouring the NM group* | Could not be calculated |
| Kurt et al. (2020) | **STATIC BALANCE**  **Ellipse area (mm^2^)**  -157.44 (-403.54; 88.66)  **COP average velocity (mm/sn)**  -1.05 (-3.45; 1.35)  **GAIT PARAMETERS**  **Cadence (step/min)**  -3.13 (-9.62; 3.36)  **Double support phase (%)**  -0.01 (-2.49; 2.47)  **Step length (cm)**  2.33 (-6.10; 10.76)  **Stride time (sn)**  -0.06 (-0.16; 0.04)  **Velocity (m/s)**  -0.02 (-0.38; 0.34) | **STATIC BALANCE**  **Ellipse area**  p = 0.20  **COP average velocity**  p = 0.38  **GAIT PARAMETERS**  **Cadence**  p = 0.33  **Double support phase**  p = 0.99  **Step length**  p = 0.57  **Stride time**  p = 0.26  **Velocity**  p = 0.91 | **STATIC BALANCE**  **Ellipse area**  ES = -0.40 [95%-CI -1.02; 0.21]  **COP average velocity**  ES = -0.27 [95%-CI -0.89; 0.33]  **GAIT PARAMETERS**  **Cadence**  ES = -0.30 [95%-CI -0.92; 0.31]  **Double support phase**  ES = -0.00 [95%-CI -0.61; 0.60]  **Step length**  ES = 0.17 [95%-CI -0.43; 0.78]  **Stride time**  ES = -0.34 [95%-CI -0.96; 0.27]  **Velocity**  ES = -0.01 [95%-CI -0.62; 0.59] |

Table 2 – Results of individual trials included in the qualitative synthesis (Neck pain)

| Study ID | Between-group difference (95% CI) | p-value (of the between-group difference) | Effect size (95% CI) |
| --- | --- | --- | --- |
| Masullo (2018) | There was a significant difference between groups only for pain intensity (values could not be calculated). | **Pain intensity**  p = 0.044*  *favouring the NM group*  **Functional status**  p > 0.05  **Pressure Pain Threshold**  p > 0.05 | Could not be calculated |
| Gupta et al. (2012) | There were significant differences between groups for pain intensity, functional status, and passive range of elbow extension in the ULNT 1 position (values could not be calculated). | **Pain intensity**  p < 0.05*  *favouring the NM group*  **Functional status**  p < 0.05*  *favouring the NM group*  **Passive elbow extension ROM in the ULNT 1 position**  p < 0.05*  *favouring the NM group* | Could not be calculated |
| Allison et al. (2002) | There was a significant difference between groups only for pain intensity (values could not be calculated). | **Pain intensity**  p < 0.05*  *favouring the NM group*  **Functional status**  p > 0.05 | Could not be calculated |

Table 3 – Results of individual trials included in the qualitative synthesis (Lateral epicondylitis)

| Study ID | Between-group difference (95% CI) | p-value (of the between-group difference) | Effect size (95% CI) |
| --- | --- | --- | --- |
| Yilmaz et al. (2022) | **Wrist flexion ROM (goniometer)**  6.19 (1.41; 10.96)*  *favouring the NM group*  **Wrist extension ROM**  1.47 (-3.37; 6.31)  **Wrist radial deviation ROM**  -0.83 (-2.93; 1.27)  **Wrist ulnar deviation ROM**  1.71 (-1.45; 4.87) | **Wrist flexion ROM**  p = 0.012*  *favouring the NM group*  **Wrist extension ROM**  p = 0.54  **Wrist radial deviation ROM**  p = 0.42  **Wrist ulnar deviation ROM**  p = 0.27 | **Wrist flexion ROM**  ES = 0.89 [95%-CI 0.19; 1.58]*  *favouring the NM group*  **Wrist extension ROM**  ES = 0.20 [95%-CI -0.45; 0.87]  **Wrist radial deviation ROM**  ES = -0.27 [95%-CI -0.93; 0.39]  **Wrist ulnar deviation ROM**  ES = 0.37 [95%-CI -0.29; 1.04] |
| Vicenzino et al. (1996) |  | **Pain intensity (NM vs. placebo)**  p < 0.05* *favouring the NM group*  **PPT (NM vs. placebo)**  p < 0.05* *favouring the NM group*  **Grip strength (NM vs. placebo)**  p < 0.05* *favouring the NM group*  **ULNT 2b ROM (NM vs. placebo)**  p < 0.05* *favouring the NM group*  **Pain intensity (NM vs. control group)**  p < 0.05* *favouring the NM group*  **PPT (NM vs. control group)**  p < 0.05* *favouring the NM group*  **Grip strength (NM vs. control group)**  p < 0.05* *favouring the NM group*  **ULNT 2b ROM (NM vs. control group)**  p < 0.05* *favouring the NM group* | **Pain intensity (NM vs. placebo)**  ES = -5.12 [95%-CI -7.02;-3.22]*  *favouring the NM group*  **Pain intensity (NM vs. control group)**  ES = 6.28 [95%-CI -8.59;-3.98]*  *favouring the NM group*  Effect sizes could not be calculated for the other variables. |

Table 4 – Results of individual trials included in the qualitative synthesis (Ankle sprain)

| Study ID | Between-group difference (95% CI) | p-value (of the between-group difference) | Effect size (95% CI) |
| --- | --- | --- | --- |
| Plaza-Manzano et al. (2016) | **Pain intensity (VAS)**  2.0 (1.3; 2.7)*  *favouring the NM group*  **Functional ankle instability (CAITS)**  5.90 (3.63; 8.16)*  *favouring the NM group*  **Muscle strength in the flexion movement (dynamometer)**  21.1 (18.9; 23.4)*  *favouring the NM group*  **Muscle strength in the extension movement (dynamometer)**  14.8 (12.3; 17.5)*  *favouring the NM group*  **Flexion ROM (goniometer)**  9.9 (7.1; 12.7)*  *favouring the NM group*  **Extension ROM**  9.5 (7.5; 11.4)*  *favouring the NM group* | p < 0.05 (for all outcomes)  *favouring the NM group* | **Pain intensity**  ES = -1.21 [95%-CI -1.78; -0.64]*  *favouring the NM group*  **Functional ankle instability**  ES = 1.39 [95%-CI 0.80; 1.97]*  *favouring the NM group*  **Muscle strength in the flexion movement**  ES = 4.84 [95%-CI 3.80; 5.88]*  *favouring the NM group*  **Muscle strength in the extension movement**  ES = 3.30 [95%-CI 2.49; 4.10]*  *favouring the NM group*  **Flexion ROM**  ES = 1.76 [95%-CI 1.14; 2.38]*  *favouring the NM group*  **Extension ROM**  ES = 2.25 [95%-CI 1.58; 2.92]*  *favouring the NM group* |

Table 5 – Results of individual trials included in the qualitative synthesis (Shoulder impingement syndrome)

| Study ID | Between-group difference (95% CI) | p-value (of the between-group difference) | Effect size (95% CI) |
| --- | --- | --- | --- |
| Akhtar et al. (2020) | **Pain intensity (VAS)**  -3.05 (-3.73; -2.36)*  *favouring the NM group*  **Functional status (UCLA score)**  9.28 (7.62; 10.93)*  *favouring the NM group* | **Pain intensity**  p < 0.001*  *favouring the NM group*  **Functional status**  p < 0.001*  *favouring the NM group* | **Pain intensity**  ES = -1.89 [95%-CI -2.41; -1.36]*  *favouring the NM group*  **Functional status**  ES = 2.48 [95%-CI 1.90; 3.07]*  *favouring the NM group* |

Table 6 – Results of individual trials (Hand osteoarthritis)

| Study ID | Between-group difference (95% CI) | p-value (of the between-group difference) | Effect size (95% CI) |
| --- | --- | --- | --- |
| Pedersini et al. (2021) | **3 months post-intervention:**  There were no significant differences between groups for pain intensity, PPTs, pinch and grip strength (values could not be calculated). | **3 months post-intervention:**  p > 0.05 (for all outcomes) | **3 months post-intervention:**  Could not be calculated |

Table 7 – Results of individual trials included in the qualitative synthesis (Plantar heel pain syndrome)

| Study ID | Between-group difference (95% CI) | p-value (of the between-group difference) | Effect size (95% CI) |
| --- | --- | --- | --- |
| Saban et al. (2014) | **Pain intensity (VAS)**  -0.1 (-0.7; 1.7)  **Functional status (CAT)**  11 (0.7; 16)*  *favouring the NM group* | **Pain intensity**  p = 0.89  **Functional status**  p = 0.02*  *favouring the NM group* | **Pain intensity**  ES = 0.03 [95%-CI -0.44; 0.50]  **Functional status**  ES = 0.55 [95%-CI 0.07; 1.04]*  *favouring the NM group* |

Table 8 – Results of individual trials included in the qualitative synthesis (Fibromyalgia)

| Study ID | Between-group difference (95% CI) | p-value (of the between-group difference) | Effect size (95% CI) |
| --- | --- | --- | --- |
| Torres et al. (2015) | **Pain intensity (VAS)**  -1.67 (-2.70; -0.63)*  *favouring the NM group*  **Functional status (HAQDI)**  0.35 (0.04; 0.65)*  *favouring the NM group*  **ULNT 1 ROM (goniometer)**  23.04 (1.87; 44.20)*  *favouring the NM group*  **ULNT 2a ROM (goniometer)**  36.41 (11.80; 61.01)*  *favouring the NM group*  **ULNT 2b ROM (goniometer)**  36.33 (15.38; 57.27)*  *favouring the NM group*  **ULNT 3 ROM (goniometer)**  7.63 (-14.34; 29.60)  **Slump stretching (goniometer)**  20.04 (6.72; 33.35)*  *favouring the NM group* | **Pain intensity**  p = 0.002*  *favouring the NM group*  **Functional status**  p = 0.02*  *favouring the NM group*  **ULNT 1 ROM**  p = 0.03*  *favouring the NM group*  **ULNT 2a ROM**  p = 0.004*  *favouring the NM group*  **ULNT 2b ROM**  p = 0.001*  *favouring the NM group*  **ULNT 3 ROM**  p = 0.48  **Slump stretching ROM**  p = 0.004*  *favouring the NM group* | **Pain intensity**  ES = -0.93 [95%-CI -1.53; -0.34]*  *favouring the NM group*  **Functional status**  ES = 0.67 [95%-CI 0.09; 1.25]*  *favouring the NM group*  **ULNT 1 ROM**  ES = 0.63 [95%-CI 0.05; 1.21]*  *favouring the NM group*  **ULNT 2a ROM**  ES = 0.86 [95%-CI 0.26; 1.45]*  *favouring the NM group*  **ULNT 2b ROM**  ES = 1.00 [95%-CI 0.40; 1.60]*  *favouring the NM group*  **ULNT 3 ROM**  ES = 0.20 [95%-CI -0.36; 0.76]  **Slump stretching ROM**  ES = 0.87 [95%-CI 0.28; 1.46]*  *favouring the NM group* |

Table 9 – Results of individual trials included in the qualitative synthesis (Unspecified musculoskeletal pain)

| Study ID | Between-group difference (95% CI) | p-value (of the between-group difference) | Effect size (95% CI) |
| --- | --- | --- | --- |
| Mateus et al. (2020) | **Pain intensity (NPRS)**  1.22 (-1.11; 3.55)  **Flexibility (SLR Test)**  3.54 (-5.68; 12.76)  **Balance (Tandem Stance Test)**  -0.84 (-3.11; 1.43)  **Gait velocity (4-m Gait Speed Test)**  0.02 (-0.06; 0.10)  **Mobility (Timed Up and Go Test)**  0.55 (-4.17; 5.27) | **Pain intensity**  p = 0.29  **Flexibility (SLR Test)**  p = 0.43  **Balance**  p = 0.45  **Gait velocity (4-m Gait Speed Test)**  p = 0.63  **Mobility (Timed Up and Go Test)**  p = 0.81 | **Pain intensity**  ES = 0.42 [95%-CI -0.35; 1.20]  **Flexibility**  ES = 0.31 [95%-CI -0.46; 1.08]  **Balance**  ES = -0.29 [95%-CI -1.07; 0.47]  **Gait velocity**  ES = 0.19 [95%-CI -0.58; 0.96]  **Mobility**  ES = 0.09 [95%-CI -0.67; 0.86] |
