# Supplementary material for: Effectiveness of Neural Mobilisation on Pain Intensity, Functional Status, and Physical Performance in Adults with Musculoskeletal Pain – A Systematic Review with Meta-Analysis
Source: Clin Rehabil. 2023 Nov 21;38(2):145–83. doi: 10.1177/02692155231215216 (PMC10725147; doi:10.1177/02692155231215216)
Supplement: sj-pdf-9-cre-10.1177_02692155231215216 - Supplemental material for Effectiveness of Neural Mobilisation on Pain Intensity, Functional Status, and Physical Performance in Adults with Musculoskeletal Pain – A Systematic Review with Meta-Analysis [file sj-pdf-9-cre-10.1177_02692155231215216.pdf]

Summary of findings:

Neural mobilization techniques compared to inactive and/or active control interventions in adults with low back pain

Patient or population: adults with low back pain
Setting: Clinical setting
Intervention: neural mobilization techniques
Comparison: inactive and/or active control interventions

Outcomes	Anticipated absolute effects* (95% CI)		Relative effect (95% CI)	№ of participants (studies)	Certainty of the evidence (GRADE)	Comments
	**Risk with inactive and/or active control interventions**	**Risk with neural mobilization techniques**				
Pain intensity assessed with: VAS / NPRS Scale from: 0 to 10 follow-up: range 1 session to 20 sessions	-	SMD **1.1 SD lower** (1.96 lower to 0.24 lower)	-	506 (12 RCTs)	⨁◯◯◯ Very low[a,b,c,d,e]	Neural mobilization techniques appear to have a large effect on pain intensity but the evidence is very uncertain.
Functional status assessed with: ODI / MODI / RMDQ follow-up: range 6 sessions to 20 sessions	-	SMD **1.12 SD lower** (1.85 lower to 0.39 lower)	-	465 (11 RCTs)	⨁◯◯◯ Very low[c,d,f,g,h]	Neural mobilization techniques appear to have a large effect on functional status but the evidence is very uncertain.
Flexibility assessed with: SLR Test / AKE Test / finger-to-ground distance follow-up: range 1 session to 20 sessions	-	SMD **1.11 SD higher** (0.08 lower to 2.3 higher)	-	214 (5 RCTs)	⨁◯◯◯ Very low[c,i,j]	Neural mobilization techniques appear to have no effect on flexibility but the evidence is very uncertain.

***The risk in the intervention group** (and its 95% confidence interval) is based on the assumed risk in the comparison group and the **relative effect** of the intervention (and its 95% CI).

CI: confidence interval; **SMD:** standardised mean difference

GRADE Working Group grades of evidence
High certainty: we are very confident that the true effect lies close to that of the estimate of the effect.
Moderate certainty: we are moderately confident in the effect estimate: the true effect is likely to be close to the estimate of the effect, but there is a possibility that it is substantially different.
Low certainty: our confidence in the effect estimate is limited: the true effect may be substantially different from the estimate of the effect.
Very low certainty: we have very little confidence in the effect estimate: the true effect is likely to be substantially different from the estimate of effect.

Explanations

a. Eight of the ten RCTs were classified at high risk of bias; the two qRCTs were classified at critical risk of bias.
b. There is a high heterogeneity between studies (large variation effect, confidence intervals do not overlap, statistical test for heterogeneity is $p < 0.01$, and the I2 is large). Even in the subgroup analyses performed, heterogeneity remains high and significant among studies of the same subgroup.
c. All studies were conducted in adults with low back pain, but the characteristics of the condition were not homogeneous across studies. Some trials only included people with chronic pain, while others included people with acute or subacute low back pain. Regarding the intervention, the studies included NM as part of a multimodal intervention or as a single intervention, as well as sliding or tensioning techniques. Different co-interventions were included in each trial. With respect to the outcome, they measured it with an appropriate measuring instrument. However, the comparison interventions varied greatly between trials. Considering all these factors, it is not possible to guarantee the generalizability, transferability, applicability, and external validity of the results.
d. Sample size > 400 participants.
e. Visual analysis using a funnel plot is inconclusive regarding publication bias (Figure 1) and the results of the Egger and Begg tests did not reject the null hypothesis of symmetry, (t = -1.74, p-value = 0.11, intercept = 1.66; z = -1.92, p-value = 0.054, respectively). The application of the Trim and Fill Method estimated two asymmetric studies on the right side of the funnel plot (Figure 2), which adjusted the global confidence interval for a non-significant effect (effect size = -0.61, 95% confidence interval: -1.62; 0.39, z = -1.19, p-value = 0.23), showing that the results found previously are not a robust estimate of the real size and significance of the effect. The p-curve analysis did not point to any selective reporting on individual trials (Figure 3).
f. Eight of the nine RCTs were classified at high risk of bias; the two qRCTs were classified at critical risk of bias.
g. There is a high heterogeneity between studies (large variation effect, confidence intervals do not overlap, statistical test for heterogeneity is $p < 0.01$, and the I2 is large). However, in the subgroup analysis ivestigating multimodal intervention vsersus single intervention, no heterogeneity was identified for the single intervention group.
h. Visual analysis using a funnel plot is inconclusive regarding publication bias (Figure 4) and the results of the Egger and Begg tests did not reject the null hypothesis of symmetry, (t = -0.89, p-value = 0.39, intercept = 0.48; z = -1.01, p-value = 0.31, respectively). The application of the Trim and Fill Method estimated 1 asymmetric study on the right side of the funnel plot (Figure 5) which did not change the significance of the results (effect size = -0.92, 95% confidence interval: -1.69; -0.15, z = -2.34, p-value = 0.01), showing that the results found previously seem a robust estimate of the real size and significance of the effect. The p-curve analysis did not point to any selective reporting on individual trials (Figure 6).
i. Three of the four RCTs were classified at high risk of bias; the qRCT was classified at critical risk of bias.
j. There is a high heterogeneity between studies (large variation effect, confidence intervals do not overlap, statistical test for heterogeneity is $p < 0.01$, and the I2 is large).

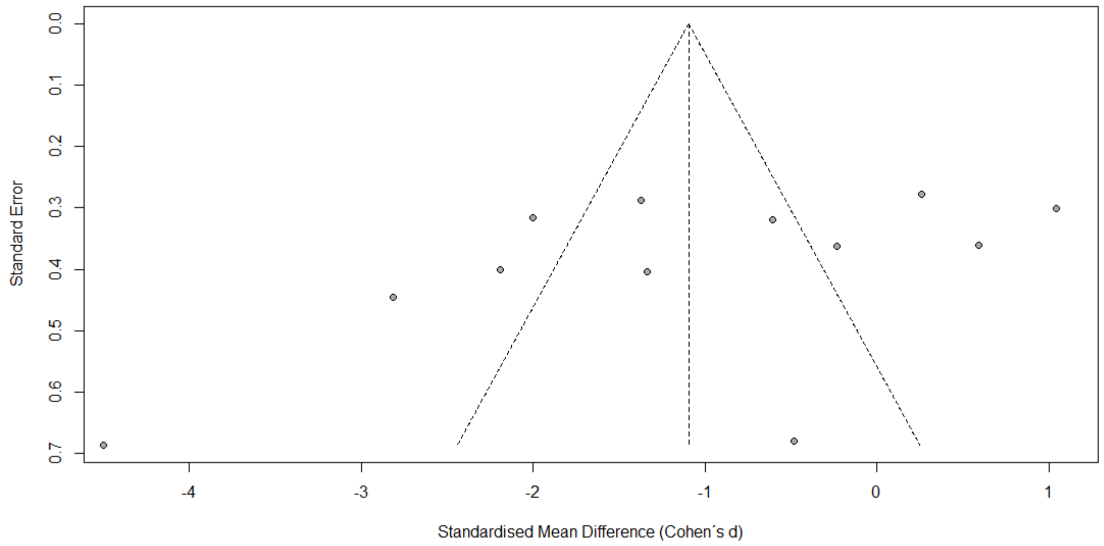

Figure 1. Funnel plot (low back pain – pain intensity)

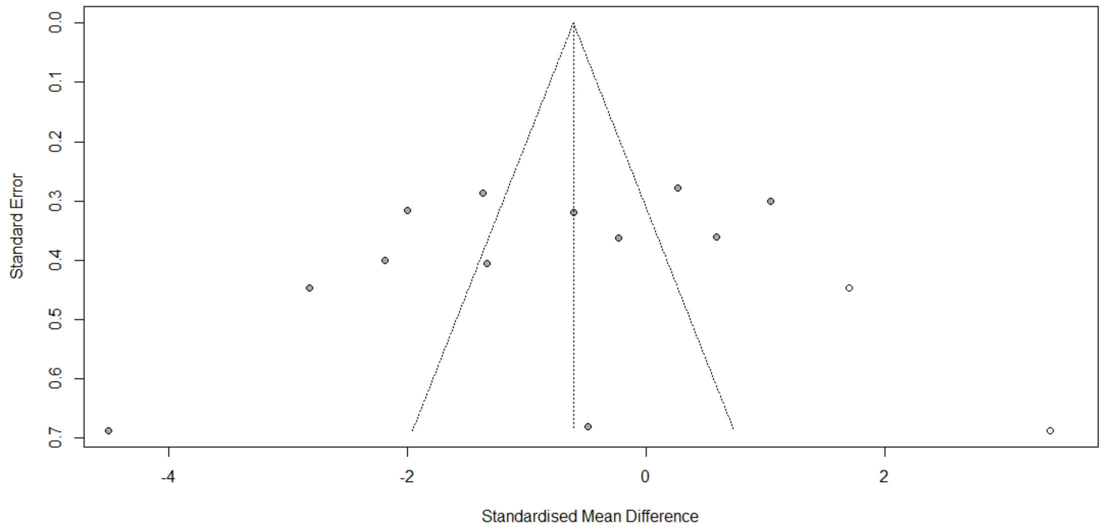

Figure 2. Funnel plot with Trim and Fill method (low back pain - pain intensity)

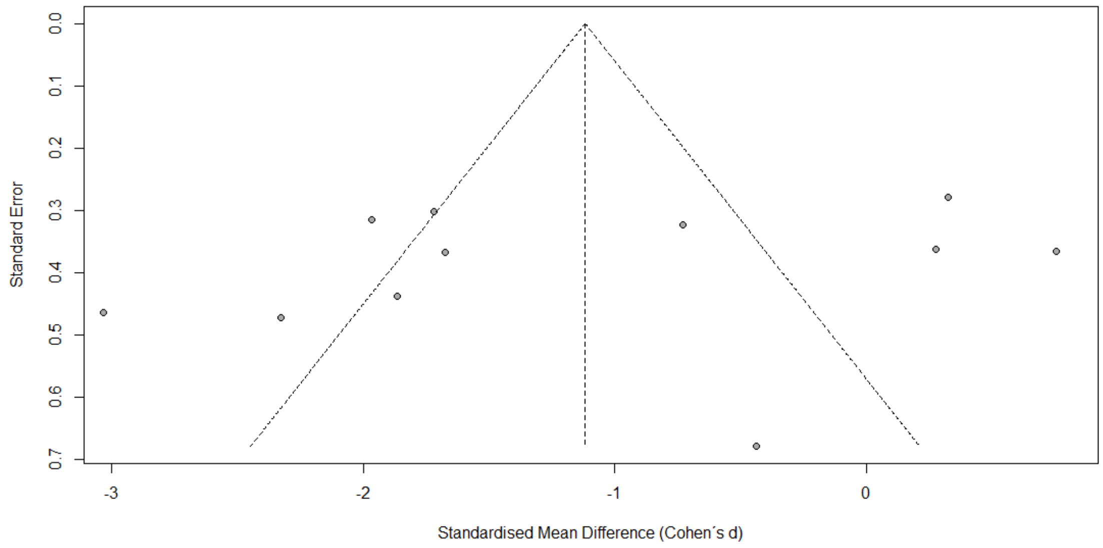

Figure 3. Funnel plot (low back pain – functional status)

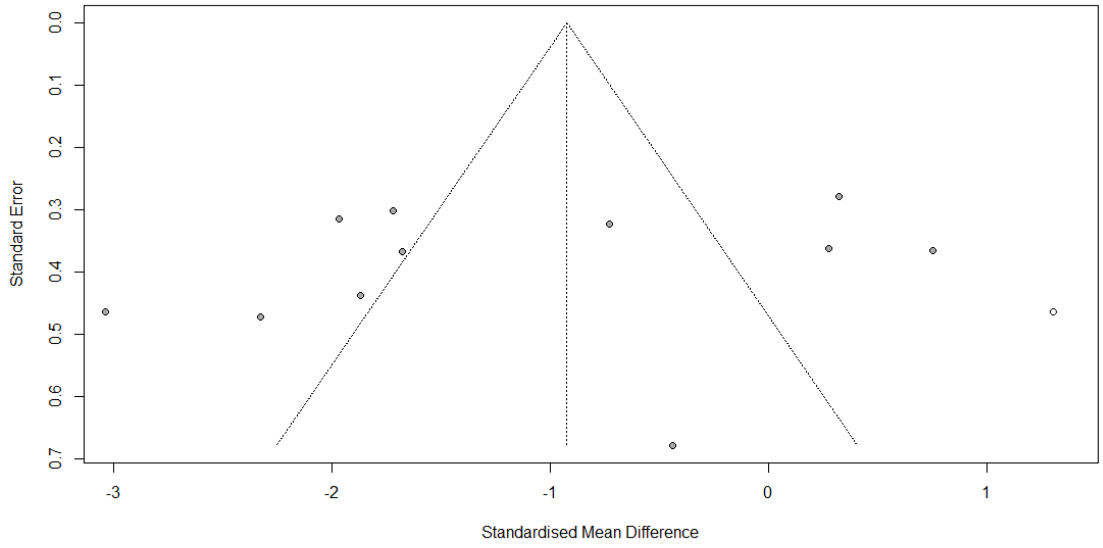

Figure 4. Funnel plot with Trim and Fill method (low back pain - functional status)

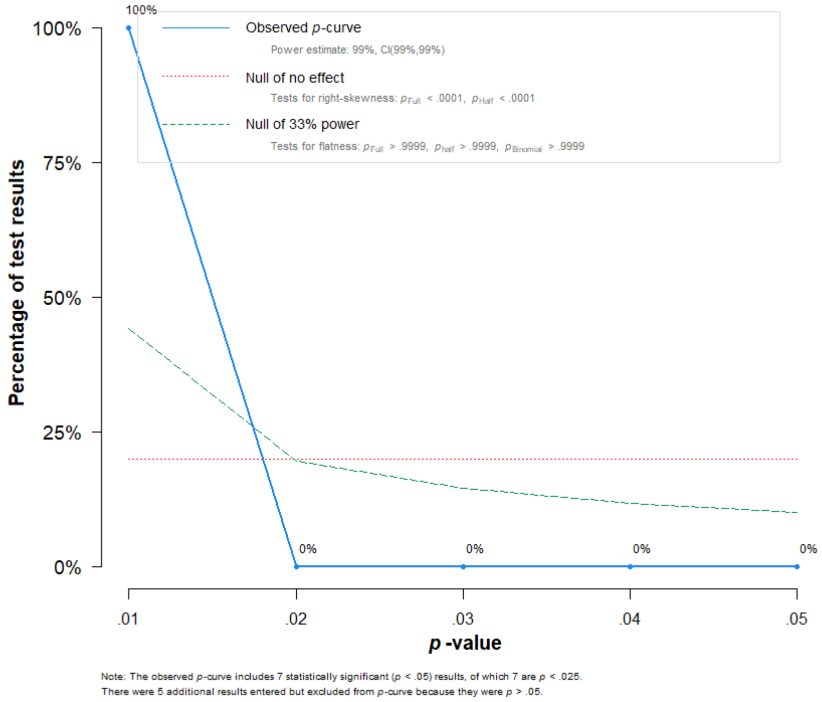

Figure 5. P-curve analysis (low back pain – pain intensity)

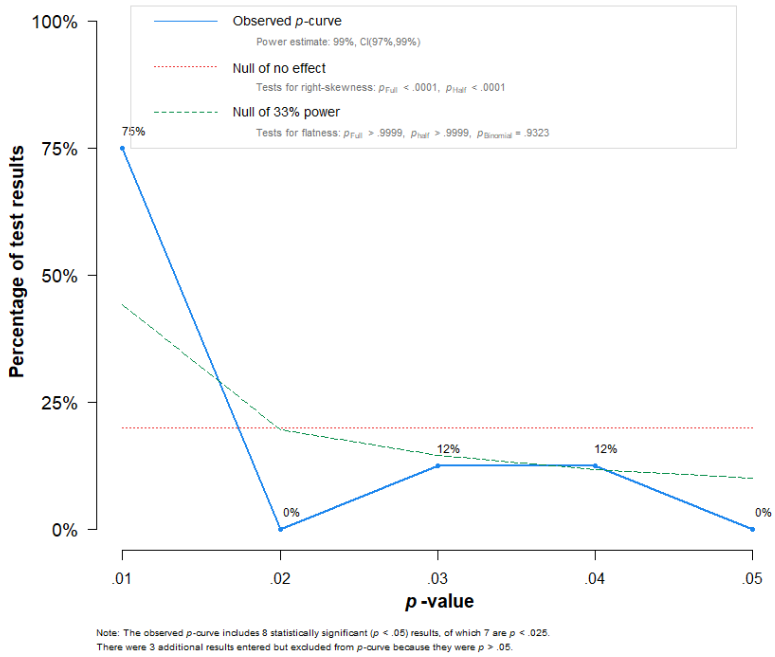

Figure 6. P-curve analysis (low back pain – functional status)

Summary of findings:

Neural mobilization techniques compared to inactive and/or active control interventions in adults with neck pain

Patient or population: adults with neck pain
Setting: Clinical setting
Intervention: neural mobilization techniques
Comparison: inactive and/or active control interventions

Outcomes	Anticipated absolute effects* (95% CI)		Relative effect (95% CI)	№ of participants (studies)	Certainty of the evidence (GRADE)	Comments
	**Risk with inactive and/or active control interventions**	**Risk with neural mobilization techniques**				
Pain intensity assessed with: VAS Scale from: 0 to 10 follow-up: range 1 session to 12 sessions	-	SMD **0.01 SD higher** (0.83 lower to 0.84 higher)	-	191 (5 RCTs)	⊕◯◯◯ Very low[a,b,c]	Neural mobilization techniques appear to have no effect on pain intensity but the evidence is very uncertain.
Functional status assessed with: NOOS / NDI follow-up: range 7 sessions to 12 sessions	-	SMD **0.07 SD lower** (1.37 lower to 1.23 higher)	-	117 (3 RCTs)	⊕◯◯◯ Very low[c,d,e,f]	Neural mobilization techniques appear to have no effect on functional status but the evidence is very uncertain.
Cervical ROM assessed with: CROM device (inclinometer)	The mean cervical ROM was **0**	**0** (0 to 0)	-	114 (3 RCTs)	⊕◯◯◯ Very low[f,g,h,i]	Neural mobilization techniques appear to have no effect on cervical ROM but the evidence is very uncertain.

***The risk in the intervention group** (and its 95% confidence interval) is based on the assumed risk in the comparison group and the **relative effect** of the intervention (and its 95% CI).

CI: confidence interval; **SMD:** standardised mean difference

GRADE Working Group grades of evidence
High certainty: we are very confident that the true effect lies close to that of the estimate of the effect.
Moderate certainty: we are moderately confident in the effect estimate: the true effect is likely to be close to the estimate of the effect, but there is a possibility that it is substantially different.
Low certainty: our confidence in the effect estimate is limited: the true effect may be substantially different from the estimate of the effect.
Very low certainty: we have very little confidence in the effect estimate: the true effect is likely to be substantially different from the estimate of effect.

Explanations

a. Four of the five RCTs were classified at high risk of bias.
b. There is a high heterogeneity between studies (large variation effect, confidence intervals do not overlap, statistical test for heterogeneity is $p < 0.01$, and the I2 is large). In the subgroup analysis performed, heterogeneity remains high and significant only for the "single intervention" group. However, for the "multimodal intervention" group, no statistical heterogeneity was identified between studies.
c. All studies were conducted in adults with neck pain, but the characteristics of the condition were not homogeneous across studies. Some trials included only people with chronic pain, while others also included people with subacute pain. Regarding the intervention, the studies included NM as part of a multimodal intervention or as a single intervention, as well as sliding or tensioning techniques. Different co-interventions were included in each trial. With respect to the outcome, they measured it with an appropriate measuring instrument. However, the comparison interventions varied greatly between trials. Considering all these factors, it is not possible to guarantee the generalizability, transferability, applicability, and external validity of the results.
d. Two of the three RCTs were classified at high risk of bias.
e. There is a high heterogeneity between studies (large variation effect, confidence intervals do not overlap, statistical test for heterogeneity is $p < 0.01$, and the I2 is large).
f. Small sample size.
g. Two of the three RCTs were classified at high risk of bias. The other was classifed with some concerns regarding the risk of bias.
h. There is a high heterogeneity between studies (large variation effect, confidence intervals do not overlap, statistical test for heterogeneity is $p < 0.01$, and the I2 is large) for flexion, extension, and lateral flexion. However, there is no heterogeneity for rotation movement.
i. All studies were conducted in adults with neck pain, but the characteristics of the condition were not homogeneous across studies. Some trials included only people with chronic pain, while others also included people with subacute pain. Regarding the intervention, two studies used sliding techniques, while the other used a tensioning technique. With regard to comparison interventions, two studies compared NM versus cervical spine mobilization, while one used a sham NM.
