# Supplementary material for: Effectiveness of Neural Mobilisation on Pain Intensity, Functional Status, and Physical Performance in Adults with Musculoskeletal Pain – A Systematic Review with Meta-Analysis
Source: Clin Rehabil. 2023 Nov 21;38(2):145–83. doi: 10.1177/02692155231215216 (PMC10725147; doi:10.1177/02692155231215216)
Supplement: sj-docx-10-cre-10.1177_02692155231215216 - Supplemental material for Effectiveness of Neural Mobilisation on Pain Intensity, Functional Status, and Physical Performance in Adults with Musculoskeletal Pain – A Systematic Review with Meta-Analysis [file sj-docx-10-cre-10.1177_02692155231215216.docx]

**Supplemental File 10 – Sensitivity analysis (Low Back Pain, Pain intensity)**

**
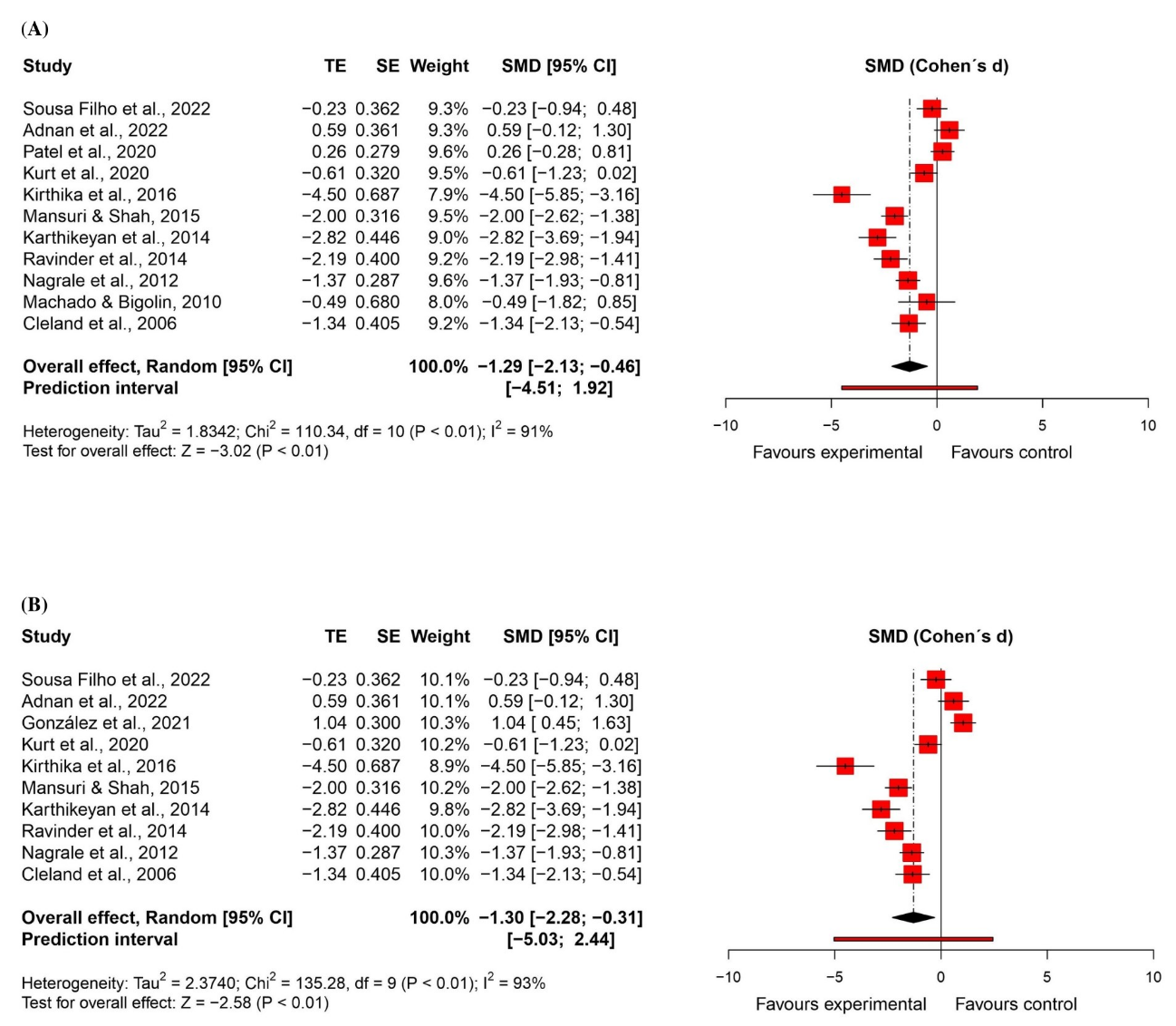
**

**A:** Low back pain (pain intensity: sensitivity analysis excluding the study of González et al., 2021); **B**: Low back pain (pain intensity: sensitivity analysis excluding studies that did not report the neural mobilization procedures used).
