# Supplementary material for: Effectiveness of Neural Mobilisation on Pain Intensity, Functional Status, and Physical Performance in Adults with Musculoskeletal Pain – A Systematic Review with Meta-Analysis
Source: Clin Rehabil. 2023 Nov 21;38(2):145–83. doi: 10.1177/02692155231215216 (PMC10725147; doi:10.1177/02692155231215216)
Supplement: sj-docx-11-cre-10.1177_02692155231215216 - Supplemental material for Effectiveness of Neural Mobilisation on Pain Intensity, Functional Status, and Physical Performance in Adults with Musculoskeletal Pain – A Systematic Review with Meta-Analysis [file sj-docx-11-cre-10.1177_02692155231215216.docx]

**Supplemental File 11**


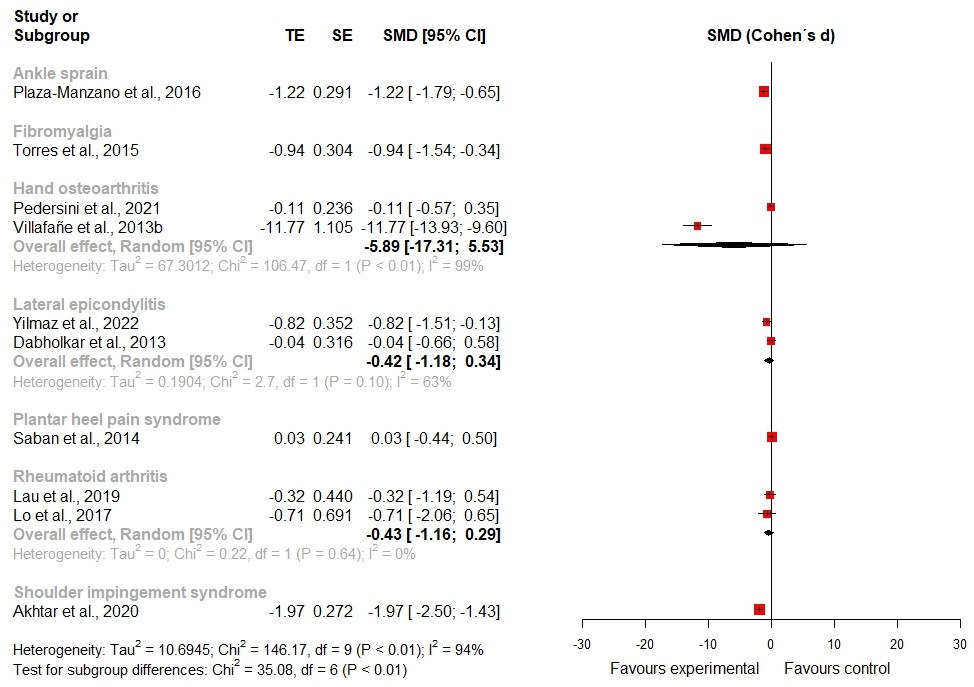


**Figure 1.** Standardized mean difference (95% CI) in the effect of neural mobilization versus other treatments on pain intensity across different musculoskeletal conditions.


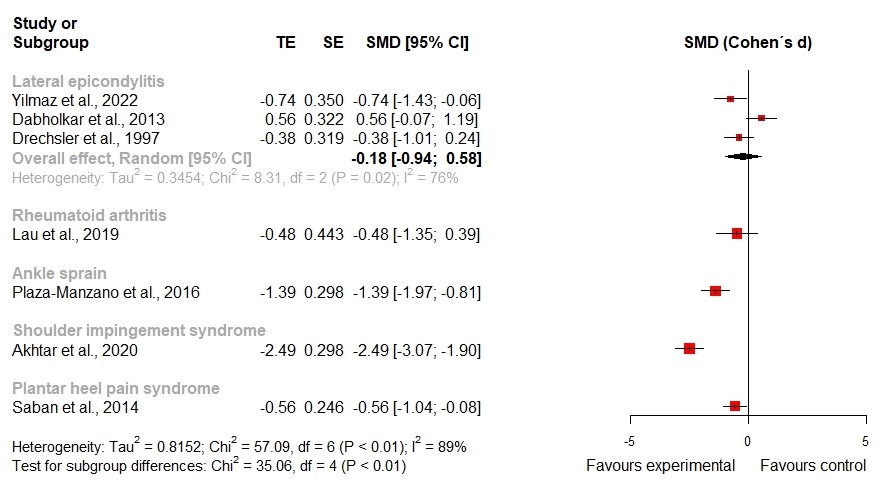


**Figure 2.** Standardized mean difference (95% CI) in the effect of neural mobilization versus other treatments on functional status across different musculoskeletal conditions.
